# Phosphorylation on serine 72 modulates Rab7A palmitoylation and retromer recruitment

**DOI:** 10.1242/jcs.262177

**Published:** 2025-01-08

**Authors:** Graziana Modica, Laura Tejeda-Valencia, Etienne Sauvageau, Seda Yasa, Juliette Maes, Olga Skorobogata, Stephane Lefrancois

**Affiliations:** ^1^ Centre Armand-Frappier Santé Biotechnologie, Institut national de la recherche scientifique, Laval, Québec H7V 1B7, Canada; ^2^ Department of Anatomy and Cell Biology, McGill University, Montreal H3A 0C7, Canada; ^3^ Centre d'Excellence en Recherche sur les Maladies Orphelines - Fondation Courtois (CERMO-FC), Université du Québec à Montréal (UQAM), Montréal H2X 3Y7, Canada

**Keywords:** Rab7A, Retromer, Palmitoylation, Endosomes, Phosphorylation, NEK7

## Abstract

Rab7A has a key role in regulating membrane trafficking at late endosomes. By interacting with several different effectors, this small GTPase controls late endosome mobility, orchestrates fusion events between late endosomes and lysosomes, and participates in the formation of and regulates the fusion between autophagosomes and lysosomes. Rab7A is also responsible for the spatiotemporal recruitment of retromer, which is required for the endosome-to-trans-Golgi network retrieval of cargo receptors such as sortilin (SORT1) and CI-MPR (also known as IGF2R). Recently, several post-translational modifications have been shown to modulate Rab7A functions, including palmitoylation, ubiquitination and phosphorylation. Here, we show that phosphorylation of Rab7A at serine 72 is important to modulate its interaction with retromer, as the non-phosphorylatable Rab7A^S72A^ mutant is not able to interact with and recruit retromer to late endosomes. We have previously shown that Rab7A palmitoylation is also required for efficient retromer recruitment. We found that palmitoylation of Rab7A^S72A^ is reduced compared to that of the wild-type protein, suggesting an interplay between S72 phosphorylation and palmitoylation in regulating the Rab7A–retromer interaction. Finally, we identify NEK7 as a kinase required to phosphorylate Rab7A to promote retromer binding and recruitment.

## Teaser

**Summary:** Rab7A is a small GTPase involved in several cellular pathways. We show that NEK7-mediated phosphorylation of Rab7A is required for its palmitoylation and, in turn, its function in retromer recruitment.

## INTRODUCTION

Late endosomes are highly dynamic organelles where the fate of proteins arriving from different cellular compartments can be decided. The small GTPase Rab7A is a principal regulator of trafficking events at late endosomes. Indeed, by engaging different effectors, Rab7A can coordinate late endosome–lysosome and autophagosome–lysosome fusion (McEwan et al., 2015), late endosome movement (van der Kant et al., 2013), positioning (Cantalupo et al., 2001; Johansson et al., 2007; Jordens et al., 2001; Rocha et al., 2009), and endosome-to-trans Golgi network (TGN) retrieval of the lysosomal sorting receptors, cation-independent mannose-6-phosphate receptor (CI-MPR, also known as IGF2R) and sortilin (SORT1) (Rojas et al., 2008; Seaman et al., 2009).

An increasing amount of data highlights the role of post-translational modifications (PTMs) as a mechanism regulating Rab7A functions (Modica and Lefrancois, 2020). Rab7A is irreversibly prenylated soon after its translation on two C-terminal cysteines (C205 and C207), and this PTM is required for its proper membrane anchoring and localization. Indeed, the Rab7^C205,207S^ mutant fails to bind to endosomal membranes and is almost completely cytosolic (Modica et al., 2017). In addition to prenylation, other modifications such as ubiquitination (Sapmaz et al., 2019; Song et al., 2016), phosphorylation (Francavilla et al., 2016; Heo et al., 2018; Malik et al., 2021; Ritter et al., 2020; Shinde and Maddika, 2016; Tudorica et al., 2024) and palmitoylation (Modica et al., 2017) have been shown to play a role in regulating Rab7A function.

We have previously shown that Rab7A can be palmitoylated on cysteines 83 and 84, and that this reversible modification is required for efficient retromer interaction, recruitment and function at endosomes (Modica et al., 2017). Retromer is an evolutionarily conserved complex composed of a trimer of the vacuolar sorting proteins Vps26, Vps35 and Vps29, which interacts with and is responsible for the endosome-to-TGN retrieval of the lysosomal cargo receptors sortilin and CI-MPR (Arighi et al., 2004; Canuel et al., 2008; Seaman, 2004). At the TGN, these receptors recognize and bind soluble lysosomal resident proteins such as cathepsin D (CTSD) and prosaposin (PSAP) and mediate their trafficking to the endosome via clathrin-coated vesicles. Once at the endosome, the more acidic pH of this compartment induces the release of cargo that is eventually trafficked to the lysosome (Bonifacino and Traub, 2003; Coutinho et al., 2012; Luzio et al., 2014). At late endosomes, the receptor is recognized and bound by retromer and retrieved back to the TGN for another round of trafficking (Arighi et al., 2004; Seaman, 2004). Impaired retromer function results in the accelerated lysosomal degradation of CI-MPR and sortilin, and dysfunction of lysosomes (Arighi et al., 2004; Yasa et al., 2020). Rab7A is required for retromer recruitment to endosomes, as downregulation or knockout of this small GTPase results in a significant displacement of retromer from the membrane to the cytosol (Modica et al., 2017; Rojas et al., 2008; Seaman et al., 2009). Palmitoylation regulates the ability of Rab7A to efficiently bind retromer, as, in Rab7 knockout (Rab7^KO^) HEK293 cells rescued with non-palmitoylatable Rab7 (Rab7^C83,83S^), retromer is not efficiently recruited to endosomal membranes (Modica et al., 2017).

Rab7A can be phosphorylated on at least two sites, tyrosine 183 (Y183) and serine 72 (S72). Y183 phosphorylation is mediated by Src kinase and inhibits the ability of Rab7A to interact with its effector RILP (Lin et al., 2017). Several kinases can mediate S72 phosphorylation, thereby regulating different Rab7A functions. Leucine rich repeat kinase 1 (LRRK1) has been shown to phosphorylate Rab7A on S72 (Malik et al., 2021), leading to an increased interaction with RILP (Hanafusa et al., 2019), whereas transforming growth factor-β (TGF-β)-activated kinase 1 (TAK1, also known as MAP3K7)-mediated S72 phosphorylation is required for endosomal maturation (Babur et al., 2020). TANK-binding kinase 1 (TBK1)-mediated phosphorylation of Rab7A on S72 regulates mitophagy (Heo et al., 2018) by inhibiting the Rab7A–rubicon (RUBCN) interaction and favouring the Rab7A–pacer (RUBCNL) interaction (Tudorica et al., 2024). Moreover, a recent report found that TBK1 phosphorylation of Rab7A S72 also promotes the activation of mTORC1 (Talaia et al., 2024).

In this work, we investigate the interplay between phosphorylation of S72 and palmitoylation of C83 and C84 in modulating the ability of Rab7A to interact with retromer. We show that the non-phosphorylatable Rab7A (Rab7A^S72A^) mutant does not efficiently interact with retromer and fails to rescue retromer membrane recruitment when expressed in Rab7^KO^ HEK293 cells. This phenotype recapitulates the behaviour of non-palmitoylatable Rab7A mutants and, indeed, we show that Rab7A^S72A^ is not efficiently palmitoylated. Finally, we identified NIMA-related kinase 7 (NEK7) as a kinase that phosphorylates Rab7A, regulating its interaction with retromer. In HEK293 cells lacking NEK7 (NEK7^KO^), we found significantly less phosphorylated Rab7A, decreased Rab7A–retromer interaction and less membrane-bound retromer.

## RESULTS

### Phosphorylation at S72 is required for the Rab7A–retromer interaction

To investigate the role of phosphorylation in modulating Rab7A–effector interactions, we first generated Rab7A mutants mimicking a constitutively phosphorylated form (phosphomimetic, Rab7A^S72E^ and Rab7A^Y183E^) or non-phosphorylatable version (phospho-null, Rab7A^S72A^ and Rab7A^Y183F^) of S72 or Y183. Recently, it was shown that Rab7A^S72E^ does not interact properly with the Rab geranylgeranyl transferase (RabGGTase), the enzyme responsible for Rab7A prenylation. This suggests that the protein does not properly localize to endosomal membranes because of the missing prenylated tail (Heo et al., 2018). Therefore, this mutant is likely non-functional, rather than a true phosphomimetic mutant, and was excluded from our study.

**Fig. 1.**
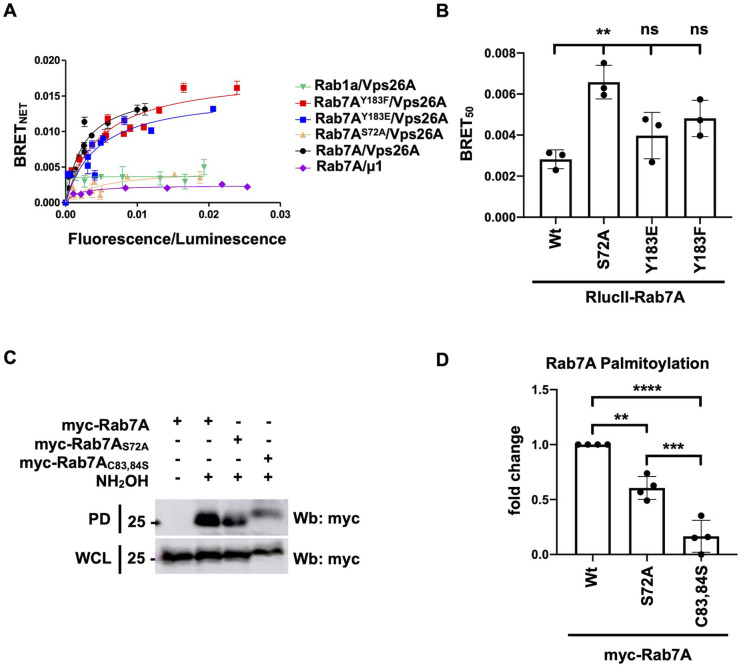
**Rab7A S72 phosphorylation regulates Rab7A–retromer interaction.** (A) HEK293 cells were transfected with a constant amount of RlucII–Rab7A wild-type (Wt, black curve), RlucII–Rab7A^S72A^ (yellow curve), RlucII–Rab7A^Y183E^ (blue curve), RlucII–Rab7A^Y183F^ (red curve) or RlucII–Rab1a (green curve), and increasing amounts of Vps26A–GFP10 or µ1–GFP10 as indicated. 48 h post-transfection, bioluminescence resonance energy transfer (BRET) analysis was performed. BRET signals are plotted as a function of the ratio between the GFP10 fluorescence over RlucII luminescence. (B) The average of the BRET_50_ value extrapolated from the BRET titration curves from three separate experiments is shown. Data are represented as mean±s.d. ns, not significant; **P<0.01; one-way ANOVA with Tukey's post hoc test. (C) Whole cell lysates (WCL) from HEK293 cells expressing wild-type myc–Rab7A, myc–Rab7A^S72A^ or myc–Rab7A^C83,84S^ were subjected to acyl-RAC analysis to determine their level of palmitoylation. NH_2_OH, hydroxylamine; PD, pull down; Wb, western blotting. (D) Quantification of three separate acyl-RAC assay experiments. Data are represented as mean±s.d. **P<0.01; ***P<0.001; ****P<0.0001; one-way ANOVA with Tukey's post hoc test.

To test the effect of phosphorylation on the ability of Rab7A to interact with its effectors, we used bioluminescence resonance energy transfer (BRET), as this technique enables us to determine protein–protein interactions in live cells with proteins expressed in their native environment. Renilla luciferase II was fused to the N-terminus of wild-type Rab7A (RlucII–Rab7A), and site directed mutagenesis was used to generate RlucII–Rab7A^S72A^, RlucII–Rab7A^Y183E^ and RlucII–Rab7A^Y183E^. We have previously shown that the addition of the RlucII tag at the N-terminus of Rab7A does not alter the ability of the protein to bind membranes or to rescue retromer recruitment in Rab7A^KO^ HEK293 cells, suggesting that this fusion protein is properly localized and functional (Modica et al., 2017). We generated BRET titration curves by co-transfecting a constant amount of RlucII–Rab7A (Fig. 1A, black curve), RlucII–Rab7A^S72A^ (Fig. 1A, yellow curve), RlucII–Rab7A^Y183E^ (Fig. 1A, blue curve) or RlucII–Rab7A^Y183F^ (Fig. 1A, red curve) with an increasing amount of the retromer subunit Vps26A or the AP-1 complex subunit µ1 tagged C-terminally with GFP10 (Vps26A–GFP10 and µ1–GFP10, respectively) (Fig. 1A, purple curve). We have previously shown that Vps26A–GFP10 is integrated into the retromer trimer and this effector efficiently binds RlucII–Rab7A but not RlucII-tagged Rab1a, a small GTPase localized to the Golgi apparatus, suggesting specificity (Modica et al., 2017; Yasa et al., 2020). By plotting the BRET_net_ values as a function of the ratio between the fluorescence emission (GFP10 emission) and the luminescence emission (RlucII emission), we calculated the BRET_50_ values from these curves. This value describes the propensity of a protein pair to interact, and the lower the value, the stronger the interaction (Kobayashi et al., 2009; Mercier et al., 2002). RlucII–Rab7A^S72A^ showed a 2.3-fold increase in BRET_50_ compared to that of wild-type Rab7A (0.0061±0.0011 and 0.0026±0.000512, respectively), suggesting that phosphorylation of S72 is required for the interaction with retromer (Fig. 1B). Although the BRET_50_ values of RlucII–Rab7A^Y183E^ and RlucII–Rab7A^Y183F^ were slightly higher than that of wild-type, they were not statistically significantly different, suggesting that the Y183 phosphorylation site does not play a role in modulating the Rab7A–retromer interaction. As a control, we tested the interaction between Rab1a and retromer (Fig. 1). RlucII–Rab1a did not interact with Vps26A–GFP10, as shown by the linear curve (Fig. 1A, green line), and RlucII–Rab7A did not interact with the AP-1 subunit µ1 (Fig. 1A, purple curve). As we observed a change in the Rab7A–retromer interaction with the Rab7A^S72A^ mutant, we tested whether this mutation affected other Rab7A–effector interactions. We generated BRET titration curves with wild-type RlucII–Rab7A or RlucII–Rab7A^S72A^ with RILP–GFP10 (Fig. S1A,B), PLEKHM1–GFP10 (Fig. S1C,D) and GFP10–FYCO1 (Fig. S1E,F). We did not find any significant changes in the BRET_50_ of these interactions, suggesting that phosphorylation at S72 is not required for the Rab7A–RILP, Rab7A–PLEKHM1 or Rab7A–FYCO1 interactions. Once again, Rab1a failed to interact with any of these known Rab7A effectors (Fig. S1A–F, green line).

We have previously shown that Rab7A palmitoylation on C83 and C84 is required for optimal retromer recruitment and function at endosomes (Modica et al., 2017). Given that Rab7A^S72A^ does not bind to retromer efficiently, we wondered whether this phenotype could be due to impaired palmitoylation of Rab7A^S72A^. We therefore performed acyl-resin assisted capture (acyl-RAC) analysis to compare the levels of palmitoylation between wild-type Rab7A, Rab7A^S72A^ and the non-palmitoylatable mutant Rab7A^C83,84S^ (Fig. 1C). We found that Rab7A^S72A^ palmitoylation was significantly reduced compared to that of wild-type Rab7A, but not as significantly as that of the non-palmitoylatable (Rab7A^C83,84S^) mutant (Fig. 1D).

### Rab7A^S72A^ is membrane bound and localized to late endosomes

To determine whether the altered Rab7A–retromer interaction we observed was due to changes in the membrane binding and localization of Rab7A, we performed a membrane separation assay as we have previously done (Modica et al., 2017; Yasa et al., 2020). HEK293 cells were transfected with myc-tagged wild-type Rab7A (myc–Rab7A), myc–Rab7A^S72A^ or myc–Rab7A^C205,207S^ (Fig. S2A). Our membrane separation was successful as the cytosolic protein tubulin was found in the soluble fraction containing the cytosol, whereas the integral membrane protein Lamp2 was found in the pellet fraction containing the membrane fraction. Although myc–Rab7A was membrane bound as expected, analysis of three independent experiments found that the Rab7A prenylation mutant (Rab7A^C205,207S^) was almost exclusively in the soluble fraction (Fig. S2B). The non-phosphorylatable mutant, Rab7A^S72A^, was also found in the pellet fraction (Fig. S2B). Although Rab7A^S72A^ was membrane bound, we wanted to exclude the possibility that the altered interaction of Rab7A^S72A^ with retromer was not due to a defect in the localization of the mutant protein. We co-transfected U2OS cells with the late endosome protein Lamp1–Cerulean and either wild-type myc–Rab7A (Fig. S2C), myc–Rab7A^S72A^ (Fig. S2D) or myc–Rab7A^C205,207S^ (Fig. S2E), and performed colocalization analysis to determine the Pearson's coefficient. Although myc–Rab7A^C205,207S^ had significantly reduced colocalization with Lamp1-Cerulean compared to wild-type myc–Rab7A, myc–Rab7A^S72A^ showed no differences in colocalizing with Lamp1–Cerulean, suggesting that the reduced binding to retromer was not due to an altered localization (Fig. S2F). Finally, we aimed to determine whether Rab7A phosphorylation on S72 was dependent on membrane anchoring and localization. We expressed myc–Rab7A, myc–Rab7A^S72A^, myc–Rab7A^Y183E^, myc–Rab7A^Y183F^, myc–Rab7A^C205,207S^ and myc–Rab7^T22N^ in HEK293 cells and performed western blotting using a well characterized antibody specific to phosphorylated S72 (pS72) on Rab7A (Malik et al., 2021; Talaia et al., 2024; Tudorica et al., 2024). In our hands, myc–Rab7A, myc–Rab7A^Y183E^ and myc–Rab7A^Y183F^ were phosphorylated on S72 (Fig. S2G), whereas myc–Rab7A^S72A^ was not (Fig. S2G). myc–Rab7A^C205,207S^ was not membrane bound due to the lack of the prenylated C-terminal tail (Fig. S2A). Although some phosphorylation was apparent on S72 of myc–Rab7A^C205,207S^, it was significantly reduced compared to that of myc–Rab7A, suggesting that Rab7A is preferentially phosphorylated on the membrane (Fig. S2G). myc–Rab7^T22N^ was not well expressed in our hands but did not appear to be phosphorylated.

### Phosphorylation on S72 is required for retromer recruitment

**Fig. 2.**
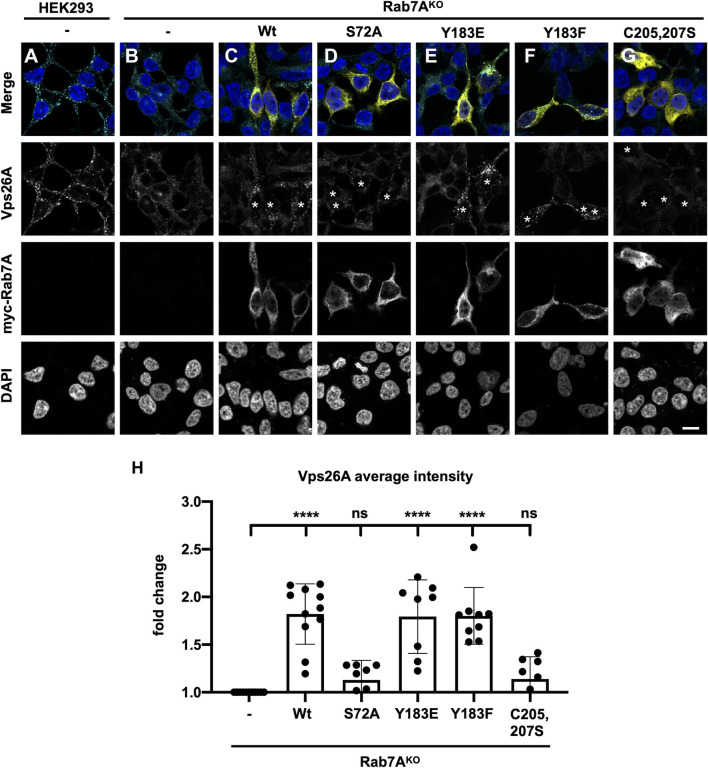
**Rab7A^S72A^ does not rescue retromer distribution in Rab7A^KO^ HEK293 cells.** (A–G) Wild-type HEK293 cells (A), Rab7A^KO^ HEK293 cells (B) and Rab7A^KO^ HEK293 cells expressing wild-type myc–Rab7A (C, asterisks), myc–Rab7A^S72A^ (D, asterisks), myc–Rab7A^Y183E^ (E, asterisks), myc–Rab7A^Y183F^ (F, asterisks) or myc–Rab7A^C205,207S^ (G, asterisks) were fixed with 4% PFA and immunostained with anti-Vps26A (cyan) and anti-myc (yellow) antibodies. DAPI was used to stain nuclei (blue). Representative images from four independent experiments are shown. Scale bar: 10 µm. (H) Quantification of Vps26A average intensity from *n*≥10 cells per condition. Values are reported as fold increase compared to Vps26A intensity in Rab7A^KO^ cells. Data are represented as mean±s.d. ns, not significant; ****P<0.0001; one-way ANOVA with Tukey's post hoc test.

As Rab7A^S72A^ palmitoylation is reduced and the mutant is unable to interact with retromer efficiently, we next asked whether S72 phosphorylation is required to efficiently recruit retromer to endosomes. We performed rescue experiments in our previously generated HEK293 Rab7^KO^ cell line (Modica et al., 2017). We determined the intensity of retromer (Vps26A) using immunofluorescence microscopy and image analysis in HEK293 wild-type (Fig. 2A), Rab7A^KO^ (Fig. 2B), or Rab7A^KO^ cells expressing either wild-type myc–Rab7A (Fig. 2C, white asterisks), myc–Rab7A^S72A^ (Fig. 2D, white asterisks), myc–Rab7A^Y183E^ (Fig. 2E, white asterisks), myc–Rab7A^Y183F^ (Fig. 2F, white asterisks) or myc–Rab7A^C205,207S^ (Fig. 2G, white asterisks). As Rab7A is required for retromer recruitment (Rojas et al., 2008; Seaman et al., 2009), the absence of Rab7A resulted in the dissociation of, but not degradation of, retromer from the membrane, resulting in a significant decrease of Vps26A puncta in Rab7A^KO^ cells (Fig. 2B,H) compared to that in parental HEK293 cells (Fig. 2A,H). The expression of wild-type myc–Rab7A (Fig. 2C,H, white asterisks), myc–Rab7A^Y183E^ (Fig. 2E,H, white asterisks) and myc–Rab7A^Y183F^ (Fig. 2F,H, white asterisks) rescued Vps26A intensity to the same extent as wild-type Rab7A (Fig. 2C,H, white asterisks). As expected, the expression of the prenylation mutant, myc–Rab7A^C205,207S^, did not rescue Vps26A intensity (Fig. 2G,H, white asterisks). Finally, the expression of myc–Rab7A^S72A^ also did not rescue Vps26A intensity (Fig. 2D,H, white asterisks). This result, combined with the BRET data demonstrating a decreased Rab7A–retromer interaction, suggests that Rab7A S72 phosphorylation is required for proper retromer recruitment to endosomes.

### Phosphorylation on S72 is not required for endocytic degradation

**Fig. 3.**
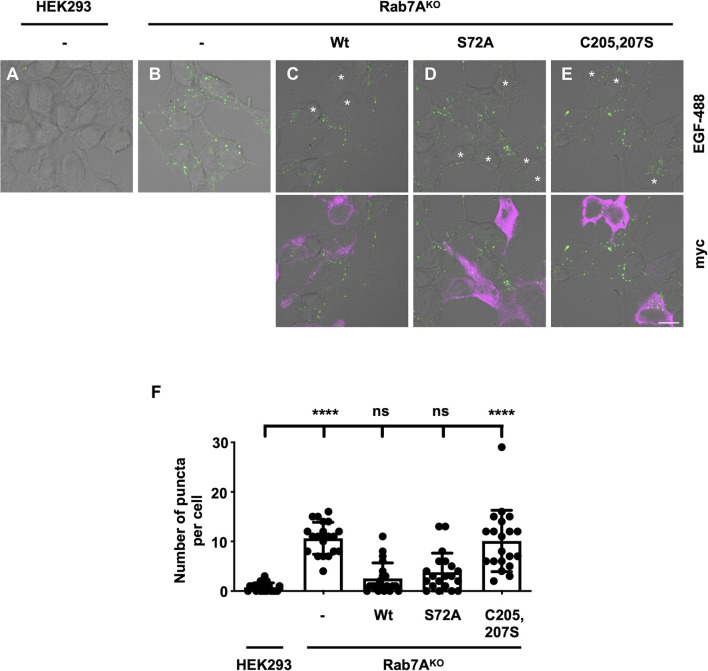
**Rab7A S72 phosphorylation is not required for EGF degradation.** (A–E) Wild-type HEK293 cells (A), Rab7A^KO^ HEK293 cells (B) and Rab7A^KO^ HEK293 cells expressing wild-type myc–Rab7A (C, asterisks), myc–Rab7A^S72A^ (D, asterisks) or myc–Rab7A^C205,207S^ (E, asterisks) were incubated with 300 ng of EGF-488 (green) for 30 min, chased for 60 min and fixed with 4% PFA. Cells were then immunostained with anti-myc (magenta) antibodies. Representative images from four independent experiments are shown. Scale bar: 10 µm. (F) Quantification of the number of EGF-488 puncta from *n*=20 cells per condition. Values are reported as the average number of puncta per cell in each condition. Data are represented as mean±s.d. ns, not significant; ****P<0.0001; one-way ANOVA with Tukey's post hoc test.

Rab7A is required for the spatiotemporal recruitment and function of retromer (Rojas et al., 2008; Seaman et al., 2009). Rab7A also plays other roles, including regulating the degradation of epidermal growth factor (EGF) and EGF receptor (EGFR) (Vanlandingham and Ceresa, 2009). To determine whether phosphorylation on Rab7A S72 was required for EGF degradation, we tested the degradation of Alexa Fluor 488-labelled EGF (EGF-488) in HEK293 cells (Fig. 3A), Rab7A^KO^ cells (Fig. 3B) and Rab7A^KO^ cells expressing wild-type myc–Rab7A (Fig. 3C, white asterisks), myc–Rab7A^S72A^ (Fig. 3D, white asterisks) or myc–Rab7A^C205,207S^ (Fig. 3E, white asterisks). Compared to wild-type cells, which had on average 0.75 puncta per cell (Fig. 3F), Rab7A^KO^ cells contained 10.65 puncta per cell (Fig. 3F), suggesting defective degradation. Expressing myc–Rab7A in Rab7A^KO^ cells rescued EGF-488 degradation, as these cells contained 2.55 puncta per cell (Fig. 3F), and expressing myc–Rab7A^S72A^ in Rab7A^KO^ also rescued degradation as we counted 3.8 puncta per cell on average (Fig. 3F). Finally, expressing myc–Rab7A^C205,207S^ in Rab7A^KO^ cells did not rescue degradation as these cells contained 10.1 puncta per cell (Fig. 3F). This suggests that phosphorylation at S72 on Rab7A is not required for endocytic degradation.

### TBK1 and TAK1 are not required for retromer recruitment

**Fig. 4.**
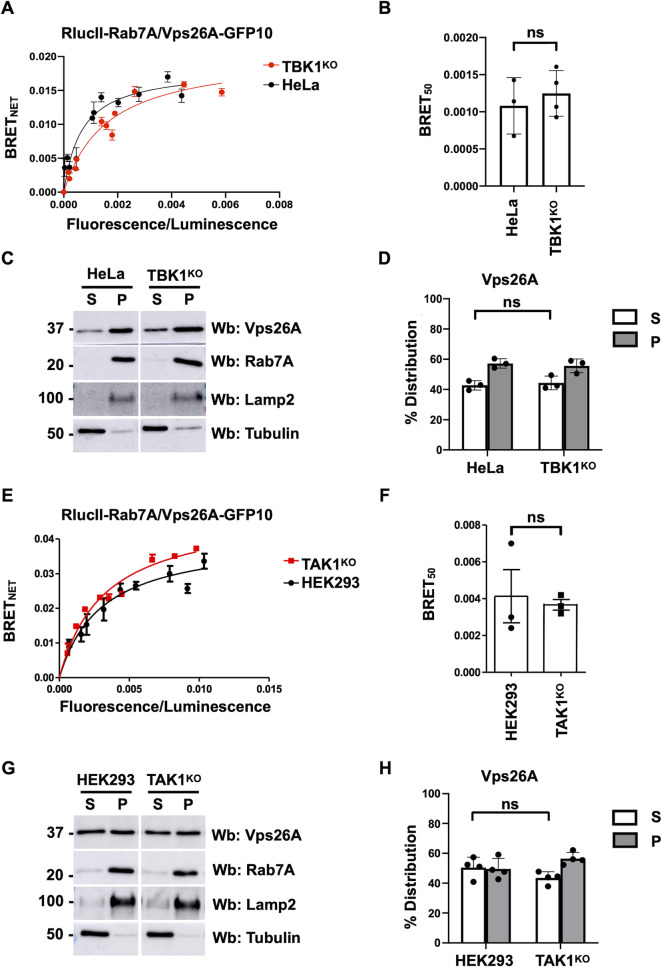
**TBK1- and TAK1-dependent phosphorylation is not required for retromer recruitment.** (A) Wild-type (black curve) or TBK1^KO^ (red curve) HeLa cells were transfected with a constant amount of RlucII–Rab7A and increasing amounts of Vps26A–GFP10. 48 h post transfection, BRET analysis was performed. BRET signals are plotted as a function of the ratio between the GFP10 fluorescence over RlucII luminescence. (B) The average of the BRET_50_ value extrapolated from the BRET titration curves from three separate experiments is shown. Data are represented as mean±s.d. ns, not significant; two-tailed unpaired *t*-test. (C) Wild-type or TBK1^KO^ HeLa cells were subjected to a membrane separation assay. The fractions were subsequently analyzed using western blotting (Wb) with anti-Vps26A, anti-Rab7A, anti-Lamp2 and anti-tubulin antibodies. Lamp2 and tubulin served as markers of the pellet fraction (P) containing membranes and the soluble fraction (S) containing the cytosol, respectively. (D) Quantification of the distribution of Vps26A from three independent membrane separation assays. Data are represented mean±s.d. ns, not significant; two-tailed unpaired *t*-test. (E) Wild-type (black curve) or TAK1^KO^ (red curve) HEK293 cells were transfected with a constant amount of RlucII–Rab7A and increasing amounts of Vps26A–GFP10. 48 h post transfection, BRET analysis was performed. BRET signals are plotted as a function of the ratio between the GFP10 fluorescence over RlucII luminescence. (F) The average of the BRET_50_ value extrapolated from the BRET titration curves from three separate experiments is shown. Data are represented as mean±s.d. ns, not significant; two-tailed unpaired *t*-test. (G) Wild-type or TAK1^KO^ HEK293 cells were subjected to a membrane separation assay. The fractions were subsequently analyzed using western blotting (Wb) with anti-Vps26A, anti-Rab7A, anti-Lamp2 and anti-tubulin antibodies. Lamp2 and tubulin served as markers of the pellet fraction (P) containing membranes and the soluble fraction (S) containing the cytosol, respectively. (H) Quantification of the distribution of Vps26A from four independent membrane separation assays. Data are represented mean±s.d. ns, not significant; two-tailed unpaired *t*-test.

TBK1 has previously been shown to phosphorylate Rab7A at S72 (Heo et al., 2018). To determine whether TBK1-dependent Rab7A phosphorylation plays a role in recruiting retromer, we used CRISPR/Cas9 to generate a TBK1 knockout (TBK1^KO^) HeLa cell line (Fig. S3A, left). Rab7A S72 phosphorylation was decreased in TBK1^KO^ compared to wild-type HeLa cells (Fig. S3A, middle), and quantification of three independent experiments showed a 45.7% decrease (Fig. S3A, right). We then used BRET to test the ability of RlucII–Rab7A and Vps26A–GFP10 to interact in TBK1^KO^ cells (Fig. 4A, red curve) compared to wild-type cells (Fig. 4A, black curve). We found no significant changes in the interaction between Rab7A and retromer in TBK1^KO^ cells compared to that in wild-type HeLa cells (Fig. 4B). We then used a membrane separation assay to compare the distribution of retromer in TBK1^KO^ and wild-type HeLa cells (Fig. 4C). We found that the distribution of the retromer subunits Vps26A (Fig. 4D) was not affected in TBK1^KO^ cells compared to that in wild-type HeLa cells. Although TBK1 can phosphorylate Rab7A on S72, TBK1 phosphorylation is not required for retromer recruitment to membranes. Recently, TAK1 has been shown to phosphorylate Rab7A (Babur et al., 2020), so we tested whether this kinase has an effect in modulating retromer recruitment. We engineered TAK1 knockout HEK293 cells (TAK1^KO^) using CRISPR/Cas9 (Fig. S3B, left). Rab7A S72 phosphorylation was reduced in TAK1^KO^ cells compared to that in wild-type HEK293 cells (Fig. S3B, middle), and quantification of four independent experiments showed a 52.5% decrease in S72 phosphorylation (Fig. S3B, right). We then used BRET to test the ability of RlucII–Rab7A and Vps26A–GFP10 to interact in TAK1^KO^ cells (Fig. 4E, red curve) compared to wild-type cells (Fig. 4E, black curve). We found no significant changes in the interaction between Rab7A and retromer in TAK1^KO^ cells compared to that in parental HEK293 cells (Fig. 4F). We next performed a membrane separation assay to compare the membrane distribution of retromer in wild-type versus TAK1^KO^ HEK293 cells (Fig. 4G). Quantification of four independent experiments found no differences in the membrane distribution of Vps26A in TAK1^KO^ cells compared to that in wild-type HEK293 cells (Fig. 4H). Once again, although TAK1 has been shown to phosphorylate Rab7A at S72, this kinase is not involved in retromer recruitment.

### NEK7 can phosphorylate Rab7A on S72 and is required for its palmitoylation

**Fig. 5.**
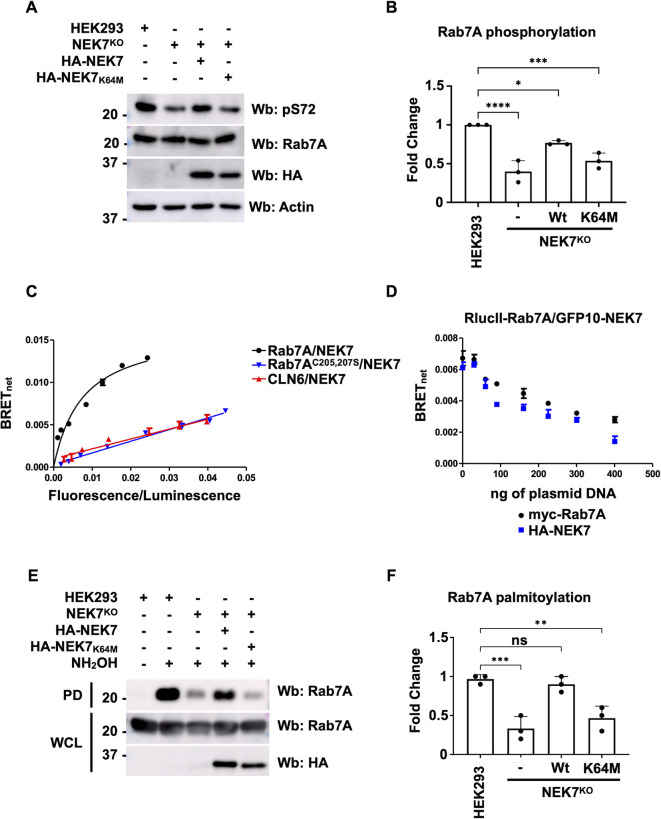
**NEK7 phosphorylates Rab7A and is required for palmitoylation.** (A) Lysates from HEK293 cells, NEK7^KO^ cells and NEK7^KO^ cells expressing either wild-type HA–NEK7 or the kinase dead mutant (HA–NEK7^K64M^) were analyzed by western blotting (Wb) with anti-phospho-S72 Rab7A (pS72), anti-Rab7A, anti-HA and anti-actin (as a loading control) antibodies. (B) Quantification of Rab7A S72 phosphorylation from three independent experiments. Data are represented as mean±s.d. *P<0.05; ***P<0.001; ****P<0.0001; one-way ANOVA with Tukey's post hoc test. (C) HEK293 cells were transfected with a constant amount of RlucII–Rab7A (black curve), RlucII–Rab7A^C205,207S^ (blue curve) or CLN6–RlucII (red curve) and increasing amounts of GFP10–NEK7. 48 h post transfection, BRET analysis was performed. BRET signals are plotted as a function of the ratio between the GFP10 fluorescence over RlucII luminescence. (D) HEK293 cells were transfected with 10 ng of RlucII–Rab7A, 150 ng of GFP10–NEK7 and 0, 50, 75, 100, 150, 250, 300 and 400 ng of myc–Rab7A (black points) or HA–NEK7 (blue points). 48 h post transfection, BRET analysis was performed. BRET signals are plotted as a function of the ratio between the GFP10 fluorescence over RlucII luminescence. (E) Whole cell lysates (WCL) from HEK293 cells, NEK7^KO^ cells and NEK7^KO^ cells expressing either wild-type HA–NEK7 or the kinase dead mutant (HA–NEK7^K64M^) were subjected to acyl-RAC to determine the level of Rab7A palmitoylation. NH_2_OH, hydroxylamine; PD, pull-down. (F) Quantification of three separate acyl-RAC assay experiments for the palmitoylation of Rab7A. Data are represented as mean±s.d. ns, not significant, **P<0.01; ***P<0.001; one-way ANOVA with Tukey's post hoc test.

A recent publication demonstrated that knockdown of NEK7 in HeLa cells resulted in the dispersal of CI-MPR into endosomes, suggesting defective retrieval of this sorting receptor (Joseph et al., 2023). This phenotype is similar to the depletion of retromer (Arighi et al., 2004; Seaman, 2004) or Rab7A (Rojas et al., 2008; Seaman et al., 2009). NEK7 is a member of the family of mammalian NIMA-related kinases (NEK proteins) and has been implicated in inflammation (He et al., 2016) and cell cycle regulation (O'Regan and Fry, 2009; Yissachar et al., 2006). As NEK7 has never been associated to phosphorylation of Rab7A, we first tested its role as a Rab7A kinase. We engineered a NEK7 knockout HEK293 cell line (NEK7^KO^) using CRISPR/Cas9 (Fig. S3C). Using this cell line, we found significantly weaker Rab7A S72 phosphorylation compared to that in wild-type cells (Fig. 5A). This decreased phosphorylation was rescued by expressing wild-type HA–NEK7 and partially rescued expressing a putative kinase-dead mutant, HA–NEK7^K64M^ (Fig. 5B). In *in vitro* assays, NEK7^K64M^ is not able to phosphorylate β-casein (O'Regan and Fry, 2009); however, in our hands, when expressed in cells, the mutant was able to partially rescue Rab7A S72 phosphorylation, although not as efficiently as wild-type NEK7. We next determined whether NEK7 could interact with Rab7A. We attempted co-immunoprecipitation using antibodies to endogenous NEK7 and Rab7A. Although immunoprecipitating with either antibody successfully isolated the target protein, we failed to isolate the other protein. The same negative result was obtained when we attempted the experiment using overexpressed tagged proteins (data not shown). The transient and potentially weak nature of such an interaction might be responsible for our inability to detect it via co-immunoprecipitation, but it might be revealed by BRET, as this technique is well suited to detect weak and transient interactions. We generated BRET titration curves by co-transfecting a constant amount of RlucII–Rab7A with an increasing amount of GFP10–NEK7 (Fig. 5C, black curve), and we were able to detect an interaction as shown by the saturating curve. We also generated BRET titration curves with the non-prenylated mutant, RlucII–Rab7A^C205,207S^ (Fig. 5C, blue curve), and CLN6–RlucII, an integral membrane protein localized to the endoplasmic reticulum (Fig. 5C, red curve), and obtained linear curves, indicating an absence of interaction. As the Rab7A prenylation mutant is almost exclusively cytosolic, these data suggest that the Rab7A–NEK7 interaction most likely occurs on the membrane. To confirm the specificity of the interaction we observed by BRET, we performed BRET competition experiments. Cells were transfected with 10 ng of RlucII–Rab7A and 150 ng of GFP10–NEK7, and excess amounts of either myc–Rab7A (Fig. 5D, black points) or HA–NEK7 (Fig. 5D, blue points). Expressing increasing amounts of either myc–Rab7A or HA–NEK7 inhibited the BRET signal between RlucII–Rab7A and GFP10–NEK7, supporting an interaction between this protein pair. As NEK7 deletion affected Rab7A phosphorylation and we demonstrated that S72 phosphorylation was required for Rab7A palmitoylation (Fig. 1C,D), we tested whether lack of NEK7 also resulted in decreased Rab7A palmitoylation. Using acyl-RAC, we determined the level of Rab7A palmitoylation in HEK293 cells, NEK7^KO^ cells and NEK7^KO^ cells expressing either HA–NEK7 or HA–NEK7^K64M^ (Fig. 5E). Compared to wild-type HEK293 cells, NEK7^KO^ cells had significantly less palmitoylated Rab7A, which was rescued by expressing HA–NEK7 and partially rescued by expressing HA–NEK7^K64M^ (Fig. 5F).

### NEK7 is required for retromer recruitment

**Fig. 6.**
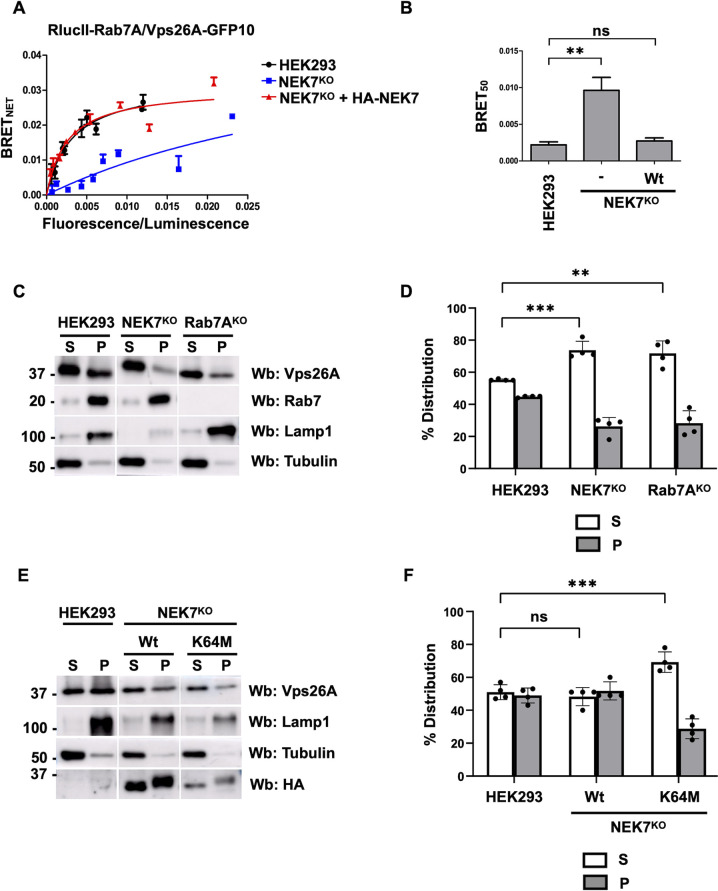
**NEK7 is required for the recruitment of retromer.** (A) Wild-type HEK293 cells (black curve), NEK7^KO^ HEK293 cells (blue curve) or NEK7^KO^ HEK293 cells expressing HA–NEK7 (red curve) were transfected with a constant amount of RlucII–Rab7 and increasing amounts of Vps26A–GFP10 to generate BRET titration curves. 48 h post transfection, BRET analysis was performed. BRET signals are plotted as a function of the ratio between the GFP10 fluorescence over RlucII luminescence. (B) The average of the BRET_50_ value extrapolated from the BRET titration curves from three separate experiments is shown. Data are represented as mean±s.d. ns, not significant; **P<0.01; one-way ANOVA with Tukey's post hoc test. (C) Wild-type, NEK7^KO^ or Rab7^KO^ HEK293 cells were subjected to a membrane separation assay. The fractions were subsequently analyzed using western blotting (Wb) with anti-Vps26A, anti-Rab7, anti-Lamp1 and anti-tubulin antibodies. Lamp1 served as a marker of the pellet fraction (P) containing membranes, and tubulin of the soluble fraction (S) containing the cytosol. (D) Quantification of the distribution of Vps26A from three independent membrane separation experiments. Data are represented as mean±s.d. ns, not significant; **P<0.01; two-way ANOVA with Dunnett's post hoc test. (E) NEK7^KO^ HEK293 cells stably expressing either HA–NEK7 or HA–NEK7^K64M^ were subjected to a membrane separation assay. The fractions were subsequently analyzed using western blotting (Wb) with anti-Vps26A, anti-Rab7, anti-Lamp1 and anti-tubulin antibodies. Lamp1 served as a marker of the pellet fraction (P) containing membranes and tubulin served as the marker for the soluble fraction (S). (F) Quantification of the distribution of Vps26A from three independent membrane separation experiments. Data are represented as mean±s.d. ns, not significant; **P<0.01; two-way ANOVA with Dunnett's post hoc test.

We next tested whether NEK7-mediated phosphorylation was required for the Rab7A–retromer interaction using BRET (Fig. 6A). We generated BRET titration curves by expressing a constant amount of RlucII–Rab7A with increasing amounts of Vps26A–GFP10 in wild-type HEK293 cells (Fig. 6A, black curve), NEK7^KO^ HEK293 cells (Fig. 6A, blue curve) or NEK7^KO^ HEK293 cells expressing HA–NEK7 (Fig. 6A, red curve). We found a 4.36-fold increase in BRET_50_ in NEK7^KO^ HEK293 cells between RlucII–Rab7A and Vps26A–GFP10 compared to that in wild-type cells (0.0096±0.0039 and 0.0022±0.0009, respectively), suggesting a significantly weaker interaction (Fig. 6B), which was rescued by expressing wild-type HA–NEK7 (0.0027±0.0009) (Fig. 6B). As retromer requires Rab7A for its membrane localization, we tested retromer membrane recruitment in NEK7^KO^ HEK293 cells (Fig. 6C). We performed a membrane separation assay to compare the distribution of retromer in wild-type, NEK7^KO^ and Rab7A^KO^ HEK293 cells (Fig. 6C). Our membrane separation was successful as the integral membrane protein Lamp1 was found in the pellet fraction, which contains membranes, whereas the cytosolic protein tubulin was found in the soluble fraction, which contains the cytosol (Fig. 6C). Quantification from four independent experiments showed that NEK7^KO^ HEK293 cells had significantly less membrane-bound retromer (26.25% in the pellet fraction) compared to wild-type HEK293 cells (44.75% in the pellet fraction), but had similar levels to Rab7^KO^ HEK293 cells (28.25% in the pellet fraction) (Fig. 6D). To determine whether retromer recruitment was dependent on NEK7 kinase activity, we expressed wild-type HA–NEK7 or the kinase-dead mutant HA–NEK7^K64M^ in our NEK7^KO^ HEK293 cells and generated stable cell lines by treating cells with the antibiotic G418. We then performed a membrane separation assay, which was successful as shown by detection of the membrane marker Lamp1 in the pellet fraction and the cytosolic marker tubulin in the soluble fraction (Fig. 6E). Quantification of three independent experiments showed that in NEK7^KO^ HEK293 cells expressing HA–NEK7, retromer distribution, as shown by Vps26A western blotting, was similar to that in wild-type HEK293 cells (51.64% in the pellet fraction), whereas the NEK7^KO^ cells expressing HA–NEK7^K64M^ had a no significant rescue (30.32% in the pellet fraction) (Fig. 6F).

### NEK7 is required to retrieve lysosomal sorting receptors and for lysosomal function

**Fig. 7.**
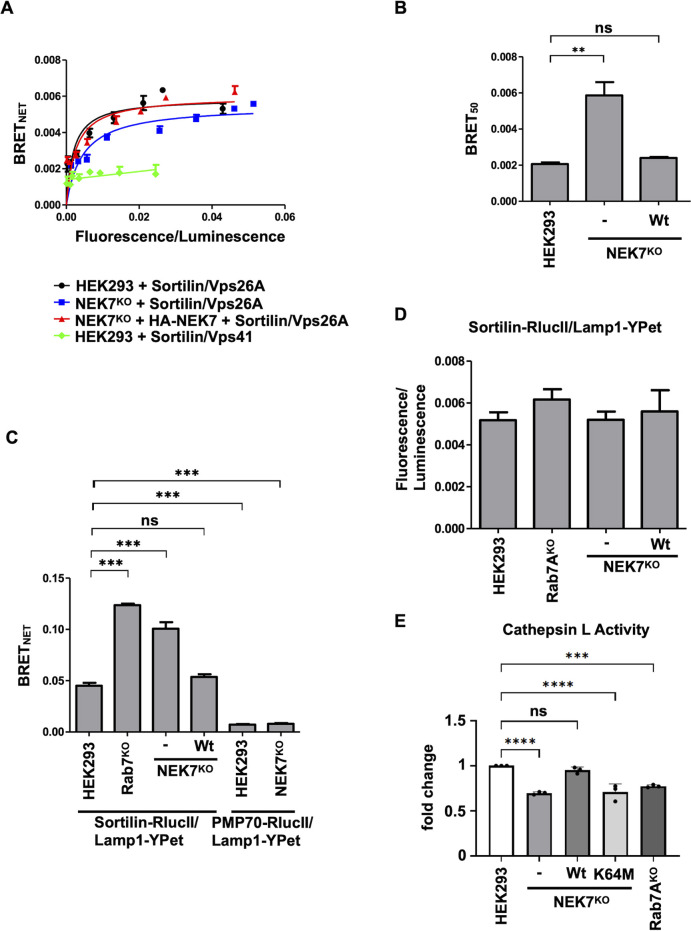
**NEK7 is necessary for efficient lysosomal function.** (A) Wild-type HEK293 cells (black and green curves), NEK7^KO^ (blue curve) or NEK7^KO^ expressing HA–NEK7 (red curve) were transfected with a constant amount of sortilin–RlucII, and increasing amounts of Vps26A–GFP10 (black, blue and red curve) or GFP10–Vps41 (green curve) to generate BRET titration curves. 48 h post transfection, BRET analysis was performed. BRET signals are plotted as a function of the ratio between the GFP10 fluorescence over RlucII luminescence. (B) The average of the BRET_50_ value extrapolated from the BRET titration curves from three separate experiments is shown. Data are represented as mean±s.d. ns, not significant; **P<0.01; one-way ANOVA with Tukey's post hoc test. (C) HEK293, Rab7A^KO^, NEK7^KO^ and NEK7^KO^ cells expressing HA–NEK7 were transfected with sortilin–RlucII or PMP70–RlucII and Lamp1–YPet. Quantification of BRET_net_ from three independent experiments is shown. Data are represented as mean±s.d. ns, not significant; ***P<0.001; one-way ANOVA with Dunnett's post hoc test. (D) HEK293, Rab7A^KO^, NEK7^KO^ and NEK7^KO^ cells expressing HA–NEK7 were transfected with sortilin–RlucII and Lamp1–YPet and the fluorescence/luminescence ratio was calculated. (E) Wild-type HEK293 cells, NEK7^KO^ cells, NEK7^KO^ cells expressing HA–NEK7 or HA–NEK7^K64M^ and Rab7A^KO^ cells were incubated with the cathepsin L Magic Red substrate for 60 min. Quantification from three independent experiments was performed. Data are represented as mean±s.d. ns, not significant; ***P<0.001; ****P<0.0001; one-way ANOVA with Dunnett's post hoc test.

On the endosomal membrane, retromer can interact with the lysosomal sorting receptor sortilin (Canuel et al., 2008). As we found less membrane-bound retromer in NEK7^KO^ HEK293 cells, we tested whether the retromer–sortilin interaction is affected in these cells. We generated BRET titration curves by expressing a constant amount of sortilin tagged to luciferase (sortilin–RlucII), with increasing amounts of Vps26A–GFP10 in wild-type HEK293 cells (Fig. 7A, black curve), NEK7^KO^ HEK293 cells (Fig. 7A, blue curve) or NEK7^KO^ HEK293 cells expressing HA–NEK7 (Fig. 7A, red curve). As a control, we also generated a BRET titration curve in wild-type HEK293 by expressing a constant amount of sortilin–RlucII, with increasing amounts of Vps41–GFP10 (Fig. 7A, green curve). No interaction was detected between sortilin and the HOPS complex subunit Vps41. Quantification of three independent experiments found a significantly reduced retromer–sortilin interaction in NEK7^KO^ HEK293 cells compared to that in wild-type HEK293 cells (0.0059±0.0013 versus 0.0021±0.0002, respectively), which was rescued by expressing HA–NEK7 (0.0024±0.0001) (Fig. 7B). As retromer did not interact efficiently with sortilin in NEK7^KO^ HEK293 cells, we would predict decreased retrieval of this cargo protein, and hence more sortilin in endolysosomes compared to that in wild-type HEK293 cells. To test this, we transfected wild-type, NEK7^KO^ and Rab7A^KO^ HEK293 cells with luciferase-tagged sortilin (sortilin–RlucII) or PMP70 (also known as ABCD3, an integral membrane protein localized to peroxisomes) (PMP70–RlucII) and an endolysosome-resident protein, Lamp1, tagged with YFP for energy transfer (YPet), a fluorescent protein derived from Venus (Nguyen and Daugherty, 2005) (Fig. 7C). We found no significant BRET_net_ signal between PMP70 and Lamp1 in either wild-type or NEK7^KO^ cells. The BRET_net_ signal from both NEK7^KO^ and Rab7A^KO^ HEK293 was significantly higher than that from wild-type cells (Fig. 7C). This is likely due to more sortilin being retained in endolysosomes, resulting in increased BRET_net_ signals, and not due to changes in the expression of either sortilin or Lamp1, as the ratios of fluorescence (Lamp1–YPet expression) over luminescence (sortilin–RlucII expression) were similar in all conditions (Fig. 7D). If sortilin is not able to efficiently be retrieved to the TGN for subsequent rounds of sorting, lysosomal activity should be disrupted. We tested the activity of cathepsin L (CTSL) using a fluorogenic substrate. The fluorescence is quenched until the enzyme, in this case, cathepsin L, cleaves the substrate releasing light. As such, a stronger fluorescence signal is interpreted as higher enzymatic activity. Compared to wild-type cells, NEK7^KO^ cells had a 30.34% decrease in cathepsin L activity, which was restored by expressing HA–NEK7 (95.1% activity compared to that in wild-type cells) but not HA–NEK7^K64M^ (29.34% decrease) (Fig. 7E). Rab7A^KO^ HEK293 cells were used as a control and showed a similar reduction in cathepsin L activity (22.67% decrease) as NEK7^KO^ cells.

## DISCUSSION

Rab7A activity at late endosomes is crucial for several pathways, including late endosome–lysosome and autophagosome–lysosome fusion (McEwan et al., 2015; van der Kant et al., 2013), late endosome movement and positioning (Cantalupo et al., 2001; Johansson et al., 2007; Jordens et al., 2001; Rocha et al., 2009), and late endosome-to-TGN protein retrieval (Rojas et al., 2008; Seaman et al., 2009). The ability of Rab7A to coordinate all these aspects of late endosome physiology is due to the capacity of this small GTPase to interact with different effectors. In this respect, PTMs can precisely modulate Rab7A function by favouring interactions with one specific effector according to cellular needs. Our previous work has shown how Rab7A palmitoylation is required to interact with and recruit retromer to endosomes. Here we characterize a further layer of regulation, where the interplay between Rab7A S72 phosphorylation and cysteine 83 and 84 palmitoylation is required for Rab7A to efficiently recruit retromer.

### Phosphorylation at S72 does not regulate Rab7 membrane association

Rab7A has been shown to be phosphorylated on at least two sites, Y183 and S72. We and others have shown that phosphorylation on these two sites is not required for Rab7 membrane association. The use of the phosphomimetic Rab7A^S72E^ could lead to the wrong interpretation of the role of S72 phosphorylation, as this mutant is mainly localized to the cytosol (Shinde and Maddika, 2016), a result that could lead to the conclusion that S72 phosphorylation acts as a switch to terminate Rab7A activity and displace the protein from the membrane. However, previous work showed that Rab7A^S72E^ interacts less with RabGGTase, the enzymes that prenylates Rab7A (Heo et al., 2018). This would suggest that the cytosolic localization of this mutant is not due its constitutive phosphorylation, but to the absence of the lipid anchor, which results in the inability of the small GTPase to stably associate to the membrane.

### NEK7-mediated Rab7 phosphorylation is required for efficient retromer function

TBK1 is related to the family of IKK kinases (I-_K_B kinase) and was first identified for its role in promoting the translocation of transcription factors during the innate immune response (Abe and Barber, 2014; Bonnard et al., 2000; Cai et al., 2014). In this context, TBK1 also has a role in activating autophagy via the phosphorylation of the autophagic adaptor optineurin (OPTN) for the lysosomal degradation of pathogens (Weidberg and Elazar, 2011). More recently the TBK1–OPTN axis has been described as crucial in maintaining cellular homeostasis by coordinating the turnover of damaged mitochondria via mitophagy and hence in maintaining cellular homeostasis (He et al., 2017; Heo et al., 2015; Richter et al., 2016). During mitophagy, TBK1 and Rab7A are independently recruited to the mitochondrial outer membrane, where TBK1 can phosphorylate Rab7A on S72, enabling the recruitment of ATG9^+^ membranes for the formation of the autophagosome (Heo et al., 2018). However, our data suggest that TBK1-mediated S72 phosphorylation is not implicated in retromer recruitment. A second kinase, TAK1, a meditator of signal transduction in response to TGF-β, has also been shown to phosphorylate Rab7A at S72 (Babur et al., 2020). Much like TBK1, we demonstrated that TAK1-dependent S72 phosphorylation is not implicated in retromer recruitment. Recent work showed that in cells lacking NEK7, CI-MPR was distributed to punctate structures, rather than being primarily localized to the Golgi apparatus (Joseph et al., 2023). This led us to investigate the role of NEK7 in Rab7A S72 phosphorylation. We found that this kinase could interact with Rab7A and regulate Rab7A S72 phosphorylation, although we did not demonstrate direct activity. Nonetheless, our data show that this kinase is required for the Rab7A–retromer interaction and retromer recruitment. How NEK7 is implicated in this process, whereas TBK1 and TAK1 are not, remains to be elucidated. Could the specific subcellular localization of the kinases themselves play a role? Although we identified a role for NEK7 in this process, other kinases could also be involved, directly or indirectly.

### S72 phosphorylation is required for efficient Rab7 palmitoylation

The modulation of protein activity via the combination of several PTMs has been shown previously for several proteins, including members of the Ras GTPase superfamily (Liu et al., 2012). Phosphorylation and palmitoylation are two major reversible PTMs used by cells to modulate the activity of proteins according to cellular needs. These PTMs can work in synergy or have opposite effects in the regulation of a protein (Charych et al., 2010; Gauthier-Kemper et al., 2014; Tian et al., 2008). As for Rab7A, we found a cooperative action of serine phosphorylation and palmitoylation in modulating its ability to interact with and recruit retromer. Our data suggest that phosphorylation on S72 is required for efficient palmitoylation; indeed, the non-phosphorylatable Rab7A^S72A^ is substantially less palmitoylated compared to wild-type Rab7A. Further supporting the need of phosphorylation for palmitoylation, Rab7A palmitoylation is significantly decreased in NEK7^KO^ HEK293 cells. According to our findings, we can speculate that serine phosphorylation facilitates the interaction of Rab7A with the palmitoylation machinery, possibly by modifying the conformation of the protein itself to favour the interaction with a still unidentified palmitoyltransferase responsible for the addition of the palmitate chain, or by preventing its interaction with thioesterases, which remove the palmitate group. This decreased palmitoylation results in less efficient interaction with retromer and could explain the inability of Rab7^S72A^ to rescue retromer endosomal recruitment in Rab7^KO^ HEK293 cells.

Previous work performed *in vitro* demonstrated an interaction between Rab7A and retromer where Rab7A would not have been palmitoylated or phosphorylated (Priya et al., 2015). Although this appears as a paradox considering the results presented here, we have previously shown that non-palmitoylatable Rab7A (Rab7A^C83,84S^) can indeed interact with retromer via co-immunoprecipitation, but not in live cells using BRET (Modica et al., 2017). In live cells, Rab7A^C83,84S^ is localized to late endosomes but does not colocalize efficiently with retromer on endosomal membrane subdomains, explaining the decreased interaction (Modica et al., 2017). This is not an issue in the co-immunoprecipitation or *in vitro* assays performed previously as these experiments occur in solution, erasing protein subcellular localization. As such, Rab7A and retromer can find one another and interact. In live cells, as the non-phosphorylatable Rab7A mutant (Rab7A^S72A^) is not palmitoylated, we hypothesize that its localization in endosomal microdomains does not match the localization of retromer, resulting in the inefficient interaction between this mutant and retromer, even though Rab7A^S72A^ is membrane bound and localized to late endosomes (Lamp1-positive compartments).

In summary, we found NEK7-dependent Rab7A S72 phosphorylation as a crucial regulator of the Rab7–retromer interaction and endosome-to-TGN trafficking pathway. Indeed, in the absence of functional NEK7 activity, the missing S72 phosphorylation hampers efficient Rab7 palmitoylation, leading to decreased retromer recruitment, inefficient retrieval of the lysosomal sorting receptors and eventually lysosomal dysfunction.

## MATERIALS AND METHODS

### Reagents, cloning and mutagenesis

Unless otherwise stated, all reagents used in this study were bought from Thermo Fisher Scientific (Ottawa, ON, Canada). The myc–Rab7A, myc–Rab7A^C83,84S^, myc–Rab7A^C205,207S^, RlucII–Rab7A, RlucII–Rab7A^C83,84S^, RlucII–Rab7A^C205,207S^, sortilin–YFP, Vps26A–GFP10, µ1–GFP10, RILP–GFP10, PLEKHM1–GFP10 and GFP10–FYCO1 constructs were previously described (Modica et al., 2017; Yasa et al., 2020). The myc–Rab7A^S72A^, RlucII–Rab7A^S72A^, myc–Rab7A^Y183E^, myc–Rab7A^Y183F^, RlucII–Rab7A^Y183E^ and RlucII–Rab7A^Y183F^ constructs were generated using site-generated mutagenesis and verified by sequencing. The sortilin–RlucII, CLN6–RlucII, PMP70–RlucII and GFP10–Vps41 constructs were generated by cloning the PCR fragment obtained from sortilin–YFP (a generous gift from Makoto Kanzaki, Tohoku University), CLN6 (MR219411, Origene Technologies, Rockville, MD, USA), PMP70–CFP (a generous gift from Frederica Theodoulou, Rothamsted Research, Harpenden, UK) or Vps41 (a generous gift from Jacques Neefjes, Leiden University Medical Center) into pcDNA3.1Hygro(+)GFP10-RlucII-st2 plasmid (a generous gift from Michel Bouvier, Université de Montreal). The following plasmids were from Addgene: pcDNA3-N-HA-NEK7 (deposited by Bruce Beutler; #75142; RRID:Addgene_75142), pcDNA3-N-HA-NEK7^K64M^ (deposited by Bruce Beutler; #75143; RRID:Addgene_75143), mCerulean-Lysosomes-20 (deposited by Michael Davidson; #55382; RRID:Addgene_55382) and YPet-Lysosomes-20 (deposited by Michael Davidson; #56636; RRID:Addgene_56636). Restriction enzymes used in this study were purchased from New England Biolabs (Danvers, MA, USA). All the mutants described in this work were generated via PCR mutagenesis using cloned PFU polymerase (Agilent Technologies, Santa Clara, CA, USA).

### Antibodies

The following mouse monoclonal antibodies were used: anti-Lamp2 [western blotting (WB): 1:500, Abcam, ab25631]; anti-myc (WB: 1:1000, IF: 1:500, Thermo Fisher Scientific, LS132500); anti-HA (WB: 1:1000, Cedarlane Labs, 901503); anti-actin (WB: 1:3000, BD Biosciences, 612657); and anti-tubulin (WB: 1:2000, Sigma-Aldrich, T9026). The following rabbit monoclonal antibodies were used: anti-Rab7A (WB: 1:1000, Cell Signaling Technology, D95F2); anti-Rab7A (pS72) (WB: 1:1000, Abcam, ab302494); anti-Lamp1 (WB: 1:1000, Cell Signaling Technology, 9091); and anti-TAK1 (WB: 1:1000, Abcam ab109526). The following rabbit polyclonal antibodies were used: anti-Vps26A (WB: 1:1000, IF: 1:500, Abcam, ab23892); anti-TBK1 (WB: 1:1000, Cell Signaling Technology, 3013); and anti-NEK7 (WB: 1:1000, Cell Signaling Technology, C34C3).

### Cell culture

All cell lines used in this study were originally obtained from American Type Culture Collection (Manassas, VA, USA) and regularly screened for contamination. HEK293, U2OS and HeLa cells were grown in Dulbecco's modified Eagle's medium (DMEM) supplemented with 10% fetal calf serum (Wisent Inc, Saint-Jean-Baptiste, QC, Canada). The Rab7^KO^ HEK293 cell line was generated using CRISPR/Cas9 approach as previously described (Modica et al., 2017). The NEK7^KO^ and TAK1^KO^ HEK293 cell lines and the TBK1^KO^ HeLa cell line were generated as previously described (Modica et al., 2017). Transfections were performed with polyethylenimine (PEI) (Thermo Fisher Scientific) as previously described (Modica et al., 2017).

### Membrane separation assay

Cell pellets were snap frozen in liquid nitrogen and thawed at room temperature (RT). Samples were then resuspended in buffer 1 [0.1 M 2-(*N*-morpholino)ethanesulfonic acid (MES)-NaOH pH 6.5, 1 mM magnesium acetate (MgAc), 0.5 mM EGTA, 200 µM sodium orthovanadate, 0.2 M sucrose] and centrifuged for 5 min at 10,000 ***g*** at 4°C. The supernatant (fraction indicated as ‘S’ in the figures) containing cytosolic proteins was collected; the remaining pellet was resuspended in buffer 2 (50 mM Tris, 150 mM NaCl, 1 mM EDTA, 0.1% SDS, 1% Triton X-100) and centrifuged for 5 min at 10,000 ***g*** at 4°C to isolate the supernatant containing membrane proteins (fraction indicated as ‘P’ in the figures). Isolated fractions were analysed via western blotting as described previously (Modica et al., 2017).

### Immunofluorescence

Immunofluorescence was performed by seeding HEK293 and U2OS cells on coverslips overnight. The following day, cells were transfected or treated as indicated in the figures. At 24 or 48 h after treatment or transfection, coverslips were washed with PBS, fixed with 4% paraformaldehyde (PFA) in PBS for 15 min at RT. PFA was removed by washing the samples three times with PBS for 5 min. Cells were blocked with 0.1% saponin and 1% BSA in PBS for 1 h at RT, followed by incubation with the primary antibody diluted in the blocking solution for 2 h at RT. Coverslips were washed three times for 5 min in PBS and incubated for 1 h at RT with secondary antibodies conjugated to either Alexa Fluor 594 or Alexa Fluor 488 in blocking solution. After one wash of 5 min in PBS, cells were incubated with DAPI, washed three times for 5 min in PBS, mounted on glass slides with Fluoromount G and sealed with nail polish.

### Acyl-RAC

Cells were lysate in TNE (150 mM NaCl, 50 mM Tris, pH 7.5, 2 mM EDTA, 0.5% Triton X-100 and protease inhibitor cocktail) supplemented with 50 mM N-ethylmaleimide (NEM) and incubated for 30 min on a rotating wheel at 4°C. Samples were centrifuged for 10 min at 10,000 ***g*** at 4°C and the collected supernatants were incubated for 2 h at RT on a rotating wheel. Samples were then precipitated overnight with two volumes of cold acetone at −20°C to remove excess NEM. After washing with cold acetone, the pellet was resuspended in binding buffer (100 mM HEPES, 1 mM EDTA, 1% SDS) with 250 mM hydroxylamine (NH_2_OH) (pH 7.5) to cleave palmitate residues off proteins. Control samples were resuspended in binding buffer containing 250 mM NaCl. When the pellet was completely resuspended, water-swollen thiopropyl sepharose 6B beads (GE Healthcare Life Sciences, Mississauga, ON, Canada) were added and samples were incubated 2 h at RT on a rotating wheel. Beads were then washed four times with binding buffer and the captured proteins were eluted with 3× sample buffer containing 100 mM dithiothreitol.

### BRET localization assay

HEK293 cells were seeded on 12-well plates overnight, followed by transfection with the indicated constructs. 48 h after transfection, cells were washed with PBS, detached with 5 mM EDTA in PBS and resuspended in 500 µl PBS. Samples were then plated in triplicate (90 μL/well) on 96-well plates (VWR Canada, Mississauga, ON, Canada). Total fluorescence was measured with an Infinite M1000 Pro plate reader (Tecan Group, Mannedorf, Switzerland), with the excitation set at 400 nm and the emission at 510 nm. The Renilla luciferase substrate coelenterazine 400a (Biotium, Fremont, CA, USA) was added to each well to a final concentration of 5 µM and the BRET signal was read after 2 min of incubation at RT. The BRET value is calculated as the ratio between the GFP10 emission (500–535 nm) over RlucII emission (370–450 nm). To calculate BRET_net_, the BRET value obtained by cells expressing only RlucII was subtracted from the BRET value registered from cells expressing both GFP10 and RlucII. To generate saturation curves, the BRET_net_ values were plotted as a function of the ratio between the GFP10 signal (fluorescence) over the RlucII signal (luminescence).

### Lysosomal activity

To determine the activity of cathepsin L, HEK293 wild-type cells, NEK7^KO^ cells, NEK7^KO^ cells expressing HA–NEK7 or HA–NEK7^K64M^, and RAB7A^KO^ HEK293 cells were collected at a concentration of 3×10^6^ cells/ml for each cell type, then transferred to 96-well black-wall plates in triplicate. Cells were then incubated with the Magic Red substrate (Bio-Rad) for 60 min at 37°C protected from light. As cells settled to the bottom, they were gently resuspended by pipetting every 10–20 min to ensure that the Magic Red substrate was evenly dispersed among all cells. The fluorescence intensity of the substrate was measured with the Tecan Infinite M1000 Pro plate reader with the excitation and emission set at 592 nm and 628 nm, respectively. The average of non-stained sample fluorescence intensities was calculated for each sample and subtracted from the fluorescence reads of the Magic Red-stained samples to eliminate background fluorescence, and signals were standardized using Hoechst stain in each sample.

### EGF-488 pulse-chase experiments

Wild-type and Rab7A^KO^ HEK293 cells were seeded on coverslips and, 24 h later, the cells were transfected with wild-type myc–Rab7A, RlucII–Rab7A^S72A^ or myc–Rab7A^C205,207S^. 24 h post transfection, cells were serum starved in Opti-MEM for 1 h, followed by a 30 min pulse of EGF-488 (Thermo Fisher Scientific) at a concentration of 300 ng/ml. Cells were then washed with PBS and fixed in 4% PFA following a 60 min chase. Cells were immunostained with anti-myc primary and Alexa Fluor 594-conjugated secondary antibodies. Cells were imaged using a Zeiss LSM 780 confocal microscope. The number of puncta per cell was counted manually (20 cells per condition for each time point).

### Image analysis and statistics

Image analysis was performed using FIJI (Schindelin et al., 2012) and the Coloc2 plugins for the colocalization analysis. Statistical analysis was performed using GraphPad Prism Version 8.2.1 (GraphPad Software, San Diego, CA, USA) and is described in the corresponding figure legends.

## Supplementary Material



10.1242/joces.262177_sup1 Supplementary information
