# Supplementary material for: Phosphorylation on serine 72 modulates Rab7A palmitoylation and retromer recruitment
Source: J Cell Sci. 2025 Jan 8;138(1):jcs262177. doi: 10.1242/jcs.262177 (PMC11828465; doi:10.1242/jcs.262177)
Supplement: Supplementary information [file joces-138-262177-s1.pdf]

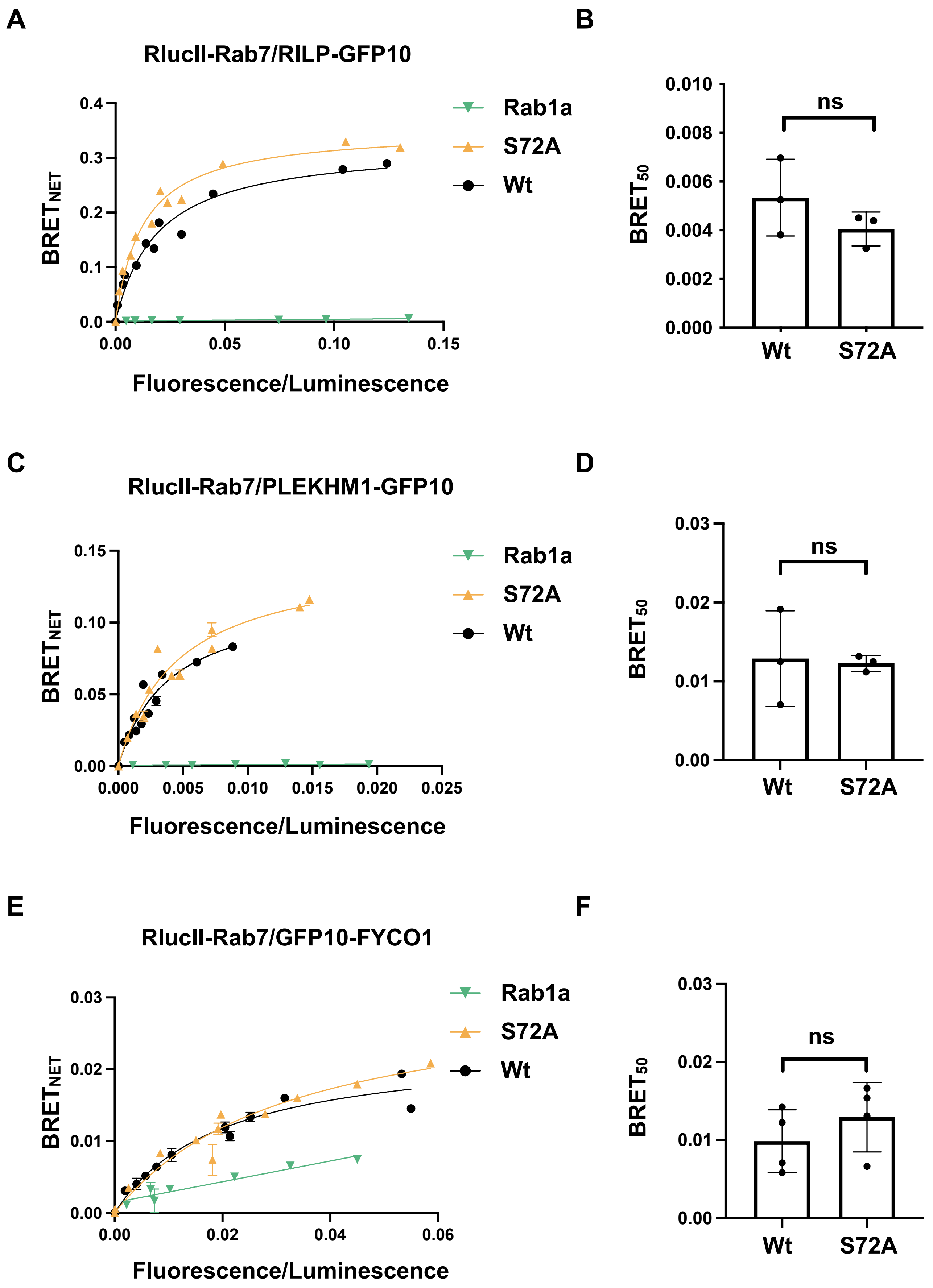

Journal of Cell Science • Supplementary information

Fig. S1 - Phosphorylation at S72 is not required for all Rab7A-effector interactions.

(A) HEK293 cells were transfected with a constant amount of RlucII-Rab7A (black curve), RlucII-Rab7A$_{S72A}$ (yellow curve), or RlucII-Rab1a (green curve) and increasing amounts of RILP-GFP10. 48 hours post-transfection BRET analysis was performed. BRET signals are plotted as a function of the ratio between the GFP10 fluorescence over RlucII luminescence. (B) The average of the BRET$_{50}$ extrapolated from the BRET titration curves from 3 separate experiments is shown. Data are represented as mean ± SD. NS, not significant; One-way ANOVA with Tukey's post-hoc test. (C) HEK293 cells were transfected with a constant amount of RlucII-Rab7A (black curve), RlucII-Rab7A$_{S72A}$ (yellow curve), or RlucII-Rab1a (green curve) and increasing amounts of PLEKHM1-GFP10. 48 hours post-transfection BRET analysis was performed. BRET signals are plotted as a function of the ratio between the GFP10 fluorescence over RlucII luminescence. (D) The average of the BRET$_{50}$ extrapolated from the BRET titration curves from 3 separate experiments is shown. Data are represented as mean ± SD. NS, not significant; One-way ANOVA with Tukey's post-hoc test. (E) HEK293 cells were transfected with a constant amount of RlucII-Rab7A (black curve), RlucII-Rab7A$_{S72A}$ (yellow curve), or RlucII-Rab1a (green curve) and increasing amounts of GFP10-FYCO1. 48 hours post-transfection BRET analysis was performed. BRET signals are plotted as a function of the ratio between the GFP10 fluorescence over RlucII luminescence. (F) The average of the BRET$_{50}$ extrapolated from the BRET titration curves from 4 separate experiments is shown. Data are represented as mean ± SD. NS, not significant; One-way ANOVA with Tukey's post-hoc test.

Journal of Cell Science • Supplementary information

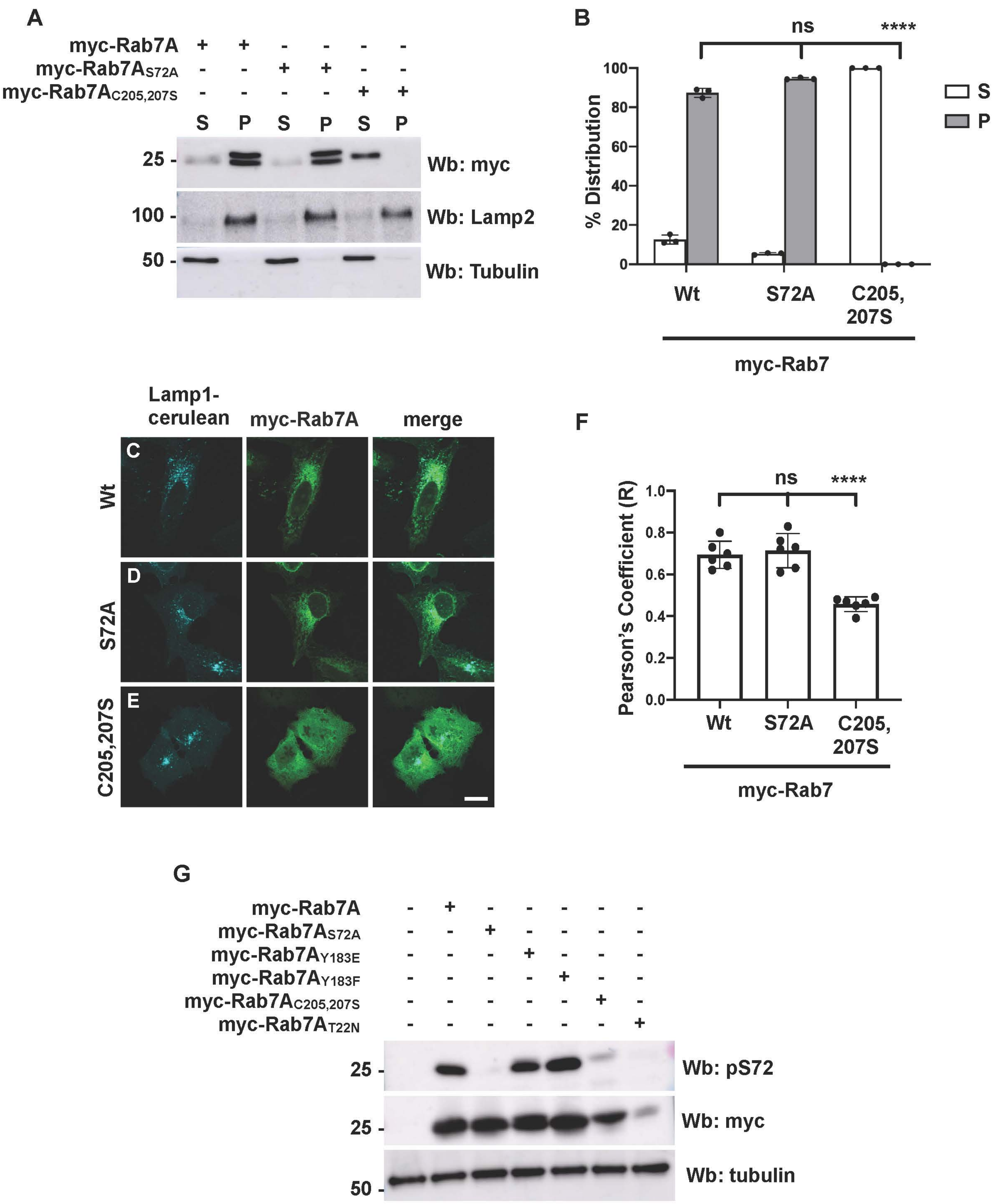

Journal of Cell Science • Supplementary information

Fig. S2. Rab7A S72 phosphorylation is not required for localization.

(A) A membrane separation assay was performed on HEK293 cells expressing myc-Rab7, myc-Rab7$_{S72A}$ or myc-Rab7$_{C205,207S}$. Western blot (Wb) was performed with anti-myc, anti-Lamp2 and anti-tubulin antibodies. Lamp2 and tubulin served as markers of the pellet fraction (P) containing membranes, and soluble fraction (S) containing the cytosol. (B) Quantification of the distribution of myc-Rab7, myc-Rab7$_{S72A}$ or myc-Rab7$_{C205,207S}$ from 3 independent experiments. Data are represented as mean ± SD. NS, not significant; **** P< 0.0001; One-way ANOVA with Tukey's post-hoc test. (C) U2OS cells were co-transfected with Lamp1-cerulean and wild-type myc-Rab7 (C), myc-Rab7$_{S72A}$ (D) or myc-Rab7C$_{83,84S}$ (E). 24 hours after transfection, cells were fixed with PFA 4% and immunostained with anti-myc antibody (green). Representative images are shown, scale bar = 10µm. (F) Pearson's correlation coefficient from n≥12 cells per condition. Data are represented as mean ± SD. , n.s. not significant, **** P < 0.0001; One-way ANOVA with Tukey's post-hoc test. (G) HEK293 cells were transfected with myc-Rab7, myc-Rab7$_{S72A}$, myc-Rab7$_{Y183E}$, myc-Rab7$_{Y183F}$, myc-Rab7$_{C205,207S}$ or myc-Rab7$_{T22N}$. 48 hours post-transfection, a Western blot (Wb) was performed using anti-phospho S72 Rab7A (pS72), anti-myc and anti-tubulin antibodies.

Journal of Cell Science • Supplementary information

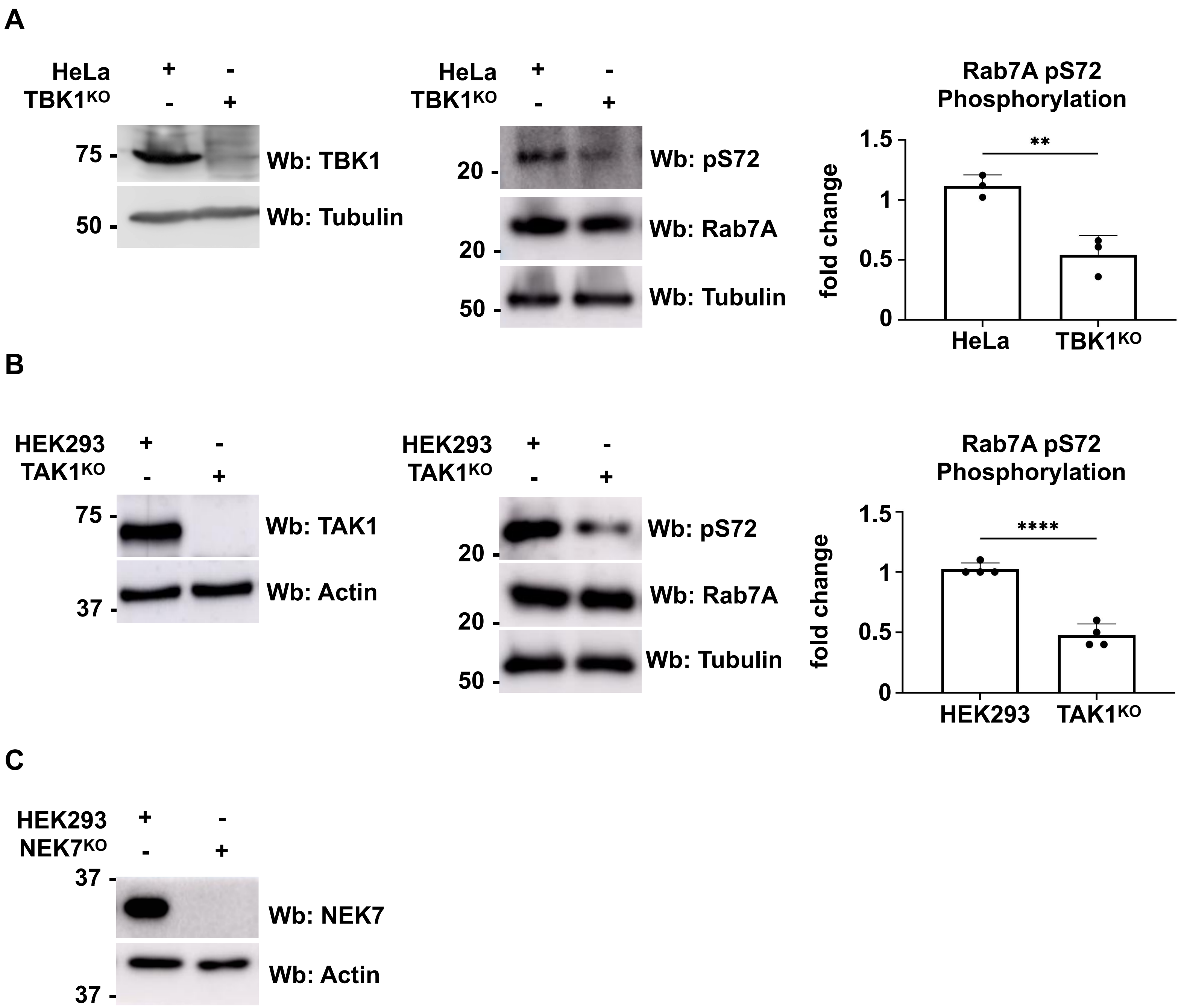

Fig. S3. Confirmation of CRISPR/Cas9 knockout

(A) Whole cells lysates from wild-type and TBK1 knockout (TBK1^{KO}) HeLa cells were run on a 12% SDS-PAGE gel, transferred to a nitrocellulose membrane, and Western blotting (Wb) was performed using anti-TBK1, anti-phospho S72 Rab7A (pS72), anti-Rab7A and anti-tubulin antibodies. Quantification of Rab7A serine 72 phosphorylation from 3 independent experiments. Data are represented as mean ± SD. ** P< 0.01; Student's t-test. (B) Whole cells lysates from wild-type and TAK1 knockout (TAK1^{KO}) HEK293 cells was run on a 12% SDS-PAGE gel, transferred to a nitrocellulose membrane, and Western blotting (Wb) was performed anti-phospho S72 Rab7A (pS72), anti-Rab7A and anti-tubulin antibodies. Quantification of Rab7A serine 72 phosphorylation from 3 independent experiments. Data are represented as mean ± SD. **** P< 0.001; Student's t-test. (C) Whole cells lysates from wild-type and NEK7 knockout (NEK7^{KO}) HEK293 cells was run on a 12% SDS-PAGE gel, transferred to a nitrocellulose membrane, and Western blotting (Wb) was performed using anti-NEK7 and anti-actin antibodies.
