# Supplementary material for: Phosphorylation on serine 72 modulates Rab7A palmitoylation and retromer recruitment
Source: J Cell Sci. 2025 Jan 8;138(1):jcs262177. doi: 10.1242/jcs.262177 (PMC11828465; doi:10.1242/jcs.262177)
Supplement: Supplementary file 1 [file joces-138-262177_review_history.pdf]

Journal of Cell Science

Phosphorylation on serine 72 modulates Rab7A palmitoylation and retromer recruitment

Graziana Modica, Laura Tejeda-Valencia, Etienne Sauvageau, Seda Yasa, Juliette Maes, Olga Skorobogata and Stephane Lefrancois
DOI: 10.1242/jcs.262177

Editor: Daniel Billadeau

Review timeline

Original submission:	4 April 2024
Editorial decision:	7 May 2024
First revision received:	28 October 2024
Accepted:	20 November 2024

Original submission

First decision letter

MS ID#: JOCES/2024/262177

MS TITLE: Phosphorylation on serine 72 modulates Rab7A palmitoylation and retromer recruitment

AUTHORS: Graziana Modica, Laura Tejeda-Valencia, Etienne Sauvageau, Seda Yasa, Juliette Maes, Olga Skorobogata, and Stephane Lefrancois
ARTICLE TYPE: Research Article

We have now reached a decision on the above manuscript.

To see the reviewers' reports and a copy of this decision letter, please go to: https://submit-jcs.biologists.org and click on the 'Manuscripts with Decisions' queue in the Author Area. (Corresponding author only has access to reviews.)

As you will see, the reviewers raise a number of substantial criticisms that prevent me from accepting the paper at this stage. All three reviewers indicate that appropriate negative and positive controls need to be included. There was also some confusion on the localization of ectopic-expressed Rab7a and mutants, localization of the phospho-mutant to endosomes, and the lack of impact on phosphorylation when NEK7 was depleted. They suggest, however, that a revised version might prove acceptable, if you can address their concerns. If you think that you can deal satisfactorily with the criticisms on revision, I would be pleased to see a revised manuscript. We would then return it to the reviewers.

Please ensure that you clearly highlight all changes made in the revised manuscript. Please avoid using 'Tracked changes' in Word files as these are lost in PDF conversion.

I should be grateful if you would also provide a point-by-point response detailing how you have dealt with the points raised by the reviewers in the 'Response to Reviewers' box. Please attend to all of the reviewers' comments. If you do not agree with any of their criticisms or suggestions please explain clearly why this is so.

Reviewer 1

© 2025. Published by The Company of Biologists under the terms of the Creative Commons Attribution License (https://creativecommons.org/licenses/by/4.0/).

Advance summary and potential significance to field

Modica et al., Phosphorylation on serine 72 modulates Rab7A palmitoylation and retromer recruitment.

In this study the authors have investigated the phosphorylation of the small GTPase Rab7a and the role of this post-translation modification (PTM) in the function of Rab7a.

The Rab7a protein is acknowledged to be a key regulator of endosomal protein sorting having a number of effectors and being linked to many important physiological processes that occur at endosomes or down-stream of endosomes.

One of the effectors of Rab7a is the retromer complex - a conserved trimer of the Vps26, Vps29 and Vps35 proteins. In mammalian cells, retromer is required for efficient endosome to Golgi retrieval and also endosome to cell surface recycling. An interaction between Rab7a and retromer was first described from studies of Rab7 in enteric amoeba and, in mammalian cells, this association was determined to be important for the targeting/recruitment of retromer to endosomes. Subsequently, the SNX3 protein has been shown to also function with Rab7a in recruitment of retromer to endosomes.

The retromer complex has been shown to be important in the pathogenesis of Parkinson’s disease and Alzheimer’s disease (AD). Indeed, SNPs and variants of the Rab7a and SNX3 genes have been linked to late onset AD and a mutation in Rab7a that causes Charcot-Marie-Tooth disease (a peripheral neuropathy) does not associate with retromer. Thus the interaction between Rab7a and retromer is an important association from both a biological perspective and also from the perspective of understanding the origin of several neurodegenerative diseases.

Comments for the author

The authors report that phosphorylation of serine 72 is mediated by the NEK protein - a serine/threonine kinase and that this PTM impacts on the function of Rab7a with respect to its association with retromer and the further modification of Rab7a by palmitoylation.

The data presented will be of interest to the field, especially those working on retromer and endosomal protein sorting but I do have suggestions below for changes and experiments that I believe will significantly strengthen the manuscript.

Major points:
1. In Figure 2, I suggest to change the presentation of the immunofluorescence image data so that the colours used are not red and green. This can be problematic for individuals who are colour blind. I would advocate for using grey-scale for the Vps26a and Rab7a images and then have a colour merged panel with colours such as yellow and cyan instead of red and green. Why does the Rab7a localisation appear largely cytosolic - is that an expression issue, ie is Rab7a expressed transiently or through stable transfection?

2. Was the imaging performed blind? ie, did the person taking the images know what condition they were viewing? This is important because it is all too easy for unconscious bias to contaminate an experiment and impact the outcome when cells are ‘selected’ for imaging because they demonstrate the ‘correct’ phenotype.

3. Is there clear evidence that the S72A Rab7a mutant is actually properly folded etc? Is it known why the S72E mutant is apparently essentially non-functional? It seems slightly strange that the two mutations would have such different effects given that the serine at position 72 is likely to be at the surface of the protein (to be accessible to the kinase) and thus not likely to be critical for folding. Does the phosphorylation state of S72 impact the ability of Rab7a to hydrolyse GTP or interact with its GTPase activating protein (GAP) - TBC1D5? The S72 residue is not that far from a critical glutamine (at position 67) that is known to be important for GTP hydrolysis via an interaction with a Rab GAP.

© 2025. Published by The Company of Biologists under the terms of the Creative Commons Attribution License (https://creativecommons.org/licenses/by/4.0/).

4. Could the graph shown in Figure 2H be redone with actual numbers rather than fold change? Also, the X-axis perhaps should include the signal obtained from imaging the ‘wildtype’ HEK293 cells which have not undergone any KO or transfection.

5. It would be helpful if the GDP-locked Rab7a mutant (T22N) was also expressed to compare its effect on retromer localisation to the S72A mutant.

6. I would suggest that Figures 3 and 4 could be moved to supplemental or else combined into a single figure as they both show data from very similar experiments investigating the ‘importance’ of other kinases that apparently phosphorylate Rab7a.

7. The use of the biochemical assay (ie supernatant versus pellet) to investigate the membrane association of retromer is a sensible approach but I am slightly surprised at the variation in the appearance of the bands and also the relative levels of membrane association between the different experiments. For example, qualitatively, the bands shown in Figure 3A look rather different from those in Figures 3E, 4A and 4E. Levels of membrane associated Vps26 seem to vary quite a lot too - approx 40% membrane associated in 3B to 60% in 3F. The bands shown for Vps26 in Figure 3E suggest that (with DMSO) ~75% is membrane associated but the graph suggests a lower number. I recognise that these biochemical assays can be sometimes trickier than a microscopy-based assay but the variation is such that forming robust conclusions about the impact of mutations etc is potentially problematic.

8. Is there any data to indicate what proportion of Rab7a is phosphorylated at S72? And also what proportion is palmitoylated? This is potentially important when the data from the Datta and Burd labs on Rab7a recruitment of retromer to endosomes is taken into consideration. Both of those labs employed in-vitro systems where the Rab7a may not be phosphorylated (or palmitoylated) but were able to show that, in vitro, Rab7a could support recruitment of retromer onto membranes. Thus, discussion of the importance of phosphorylation of S72 should incorporate findings from in-vitro systems where PTMs may not occur.

9. Why was the loss of NEK on retromer localisation (ie Vps26) not investigated using immunofluorescence microscopy - as in Figure 2? This seems like an obvious approach to take.

10. Does loss of NEK7 impact on sortillin stability? This would be predicted (and possibly expected) if sortillin is not being efficiently retrieved from endosomes to the Golgi.

11. Does mutation of S72 (or loss of NEK7) impact on the localisation of Glut1, a membrane protein that is dependent on retromer for its endosome to cell surface recycling?

Minor points:
1. Perhaps it is now appropriate to no longer describe Rab7a-positive endosomes as ‘late’ endosomes. The ‘early’ and ‘late’ terminology no longer accurately describes the complexity or dynamism of the endosomal system and leaves the reader with the impression that endosomes comes in only two flavours - early and late. In my view, it is now time for the endosomal system to be viewed as a continuum of maturing compartments which are in a constant state of flux.

2. I'm not sure why the Canuel 2008 paper is cited with respect to Rab7a coordinating retrieval from endosomes to the Golgi as that study is clearly focussed on the opposing roles of AP1 and retromer in mediating sortilin localisation. I would suggest moving that citation to more appropriate location in the text.

Reviewer 2

Advance summary and potential significance to field

General comments:

In this manuscript, Modica et al. extend on their previous work on Rab7 palmitoylation (Modica et al. JCS 2017). The authors establish a link between Rab7 phosphorylation at position S72 and

© 2025. Published by The Company of Biologists under the terms of the Creative Commons Attribution License (https://creativecommons.org/licenses/by/4.0/).

palmitoylation at positions C83/C84. They demonstrate that Rab7 with an S72A mutation is less able to interact with VPS26A consistent with their previous observations using Rab7 C83S/C84S, and identify NEK7 as a Rab7 S72 kinase.

Comments for the author

The text is well-written, and figures are clearly presented. Some conclusions are well-supported by the data however, some assays used in the manuscript require additional controls for the data to be properly interpreted; see specific points below.

Specific points:
Figure 1A, 5C, 7A, 7C – The BRET experiments require appropriate negative controls, such as non-interacting GFP- and RLuc- labelled proteins, to assess the background BRET in each experimental system. This approach was used by the authors in their previous manuscript (Modica et al. JCS 2017, Figure 5A/B).

Figure 2 – The data in this figure show a decrease in overall VPS26A intensity, however, there is still a punctate distribution in the images. Therefore, these data are more consistent with a loss of whole-cell levels of retromer, and not specific dissociation from endosomes as described in the text.

Figure 3, 4 – These data are difficult to interpret without controls to show that the inhibition/knockout of TBK1 and TAK1 is working correctly. Please could the authors include blots to show decreased phosphorylation of TBK1 (Figure 3) or TAK1 (Figure 4) substrates, and what effect the drugs/knockouts have on Rab7 S72 phosphorylation?

Figure 7C – It is surprising that the authors see such a robust BRET signal for LAMP1-sortilin when these proteins are not known to interact strongly (BRET = ~0.12 compared to a maximum of ~0.03 for Rab7/VPS26A). Therefore, it is particularly important to include appropriate negative/positive controls for this BRET pair and perform a titration similar to those in Figures 1A, 5C, 6A. Furthermore, Rab7 and NEK7 knockouts could substantially alter LAMP1/sortilin abundance and subcellular localisation, so additional controls should be included to account for possible indirect effects on BRET signal.

Figure 7D – This assay also requires an appropriate negative control to assess the background signal, such as a sample without the Cathepsin L substrate or a sample with a Cathepsin L inhibitor.

Abstract/Discussion – The authors describe NEK7 as “the kinase required to phosphorylate Rab7”. However Figure 5 shows that Rab7 maintains clear S72 phosphorylation even with the NEK7 knockout, so this statement should be altered to reflect the possibility of other kinases being involved.

<u>Reviewer 3</u>

Advance summary and potential significance to field

The manuscript by Modica et al. investigates how phosphorylation of the late endosomal GTP-binding protein Rab7, specifically Serine 72, regulates Rab7 palmitoylation. The authors analyzed interaction of the phosphorylation-defective Rab7 mutant with the known interaction partner, the retromer complex. The authors have found that the “non-phosphorylatable Rab7A (Rab7AS72A) mutant does not efficiently interact with Vps26 subunit of retromer and fails to rescue retromer membrane recruitment when expressed in Rab7KO HEK293 cells. To uncover the mechanism, the authors analyzed the palmitoylation status of the phospho-defective Rab7 mutant and found that “Rab7AS72A is not efficiently palmitoylated." Neither TANK-binding kinase 1 (TBK1) nor TAK1 kinases (that have been shown to phosphorylate Rab7A) were found to regulate retromer membrane localization.

© 2025. Published by The Company of Biologists under the terms of the Creative Commons Attribution License (https://creativecommons.org/licenses/by/4.0/).

However, the authors identified NEK7 kinase as a regulator of Rab7 phosphorylation and retromer localization. The authors also showed that NEK7 KO cells had reduced interaction between retromer and one of its cargos, Sortilin. Sortilin localization was likely more endolysosomal in NEK7 KO cells.

Comments for the author

The manuscript presents an interesting finding on regulation of Rab7, which is a key regulator of late endosomal trafficking. The study identifies NEK7 as one of the kinases that mediates Rab7 phosphorylation at S72 and thus acts as a regulator of Rab7 function.

The authors show that Rab7AS72A interaction with retromer subunit Vps26 is reduced, and Vps26/retromer is displaced from vesicles. The mechanism behind the reduced effector binding of Rab7AS72A is unclear from the presented data. Is this serine residue conserved in other Rab proteins and whether phosphorylation of serine residue at this position reported for other Rabs? Can the authors speculate from previous structural data on Rab-effector complexes, how this phosphorylation would change effector binding?

The authors should assess whether there is any change in GTP binding of Rab7AS72A as compared to the WT protein. Is Rab7AS72A defective in binding to only retromer or to other Rab7 effectors, such as RILP, ORP1L and PLEKHM1? Any change in GTP binding would generally also result in a change in Rab's membrane localization.

The authors should also analyse the localization and binding of Vps35 subunit of retromer, which is the direct binding partner of Rab7 in this complex. Retromer is a trimeric complex composed of the Vps35-Vps26 and Vps29 subunits. To conclude that the complete retromer complex is displaced from membranes, the authors should also show localization of the other subunits in the presence of Rab7 WT and mutants and interaction of the Rab7 S72A mutant with the complete retromer complex.

The authors show that the phosphorylation-defective Rab7 mutant (Rab7AS72A) continues to localize to membranes, however, the palmitoylation-defective Rab7C83,84S mutant translocates into the cytosol (Fig. S1 C–E). The author should provide an explanation that if Rab7AS72A is partially defective in palmitoylation then how its membrane localization remain unchanged?

From figure 5A, it appears that a significant proportion of total Rab7 is phosphorylated at S72 under endogenous conditions, and that this is reduced upon Nek7 knockout. Where is this phosphorylation likely to occur, i.e., in the cytosol or on late endocytic membranes? Is Nek7 localized to late endosomes? The authors should also show the specificity of the anti-phospho Rab7 antibody used in Figure 5A

The authors have not analysed significance of Rab7 phosphorylation in late endosome/lysosome positioning and lysosomal cargo degradation. To better assess the significance of phosphorylation at this residue, the authors should assess whether Rab7AS72A can rescue the cargo trafficking function in the absence of Rab7, such as for cargo destined for degradation like EGFR or LDL degradation.

Further, Rab7 regulates late endosome positioning in the perinuclear region by recruiting RILP-dynein-dynactin complex on late endosomes. Does expression of Rab7AS72A affect late endosome/lysosome positioning?

The authors show that NEK7 mediates Rab7 phosphorylation at S72. Is there endocytic cargo degradation defects in NEK7 KO cells and whether the same can be rescued by overexpression of Rab7WT?

Below, I list some of the concerns in specific figures, as well as my suggestions to strengthen the study's major conclusions:

1) Fig. 1C: The mobility of palmitoylation-defective Rab7C83,84S is shifted upward in both pulldown and WCL. The same upward mobility shift is not evident in Rab7AS72A. What is the reason behind the upward mobility shift of Rab7C83,84S?

© 2025. Published by The Company of Biologists under the terms of the Creative Commons Attribution License (https://creativecommons.org/licenses/by/4.0/).

2) The localization data shown in Figure 2 (A–G) is confusing. Myc Rab7A constructs, both WT and all the mutants, except Y183F, appear to be very cytosolic. Typically, Rab7a WT is expected to be on membrane-bound vesicles. This is not evident in Fig. 2A.

3) In the same set of panels, i.e., Figure 2 (A–G), it is unclear why Vps26 total intensity is reduced in cells lacking functional Rab7. Shouldn't it be that Vps26 shifts from vesicles to the cytosol in cells with reduced Rab7 function?

4) To strengthen the findings of Fig. 7C, the authors should also show images and measure Sortilin colocalization with LAMP1-positive compartments under different conditions.

Minor concerns:
-Pg 3 and 4: Mutant RlucII-Rab7A Y183F is miswritten as RlucII-Rab7A Y183E in the

- Fig 1B- myc Rab7 is labeled on X axis while RlucII-Rab7A constructs have been used for transfections.

-Fig S1 C, D, and E: scale bar missing

-Page 7: 10 ng- symbol is incorrect

-Page 11: Typo in Rab7A s72 phosphorylation.

-Page 13: Membrane Separation Assay- what does the statement centrifuged 5' mean?

First revision

Author response to reviewers' comments

Reviewer 1 Advance Summary and Potential Significance to Field:
Modica et al., Phosphorylation on serine 72 modulates Rab7A palmitoylation and retromer recruitment.

In this study the authors have investigated the phosphorylation of the small GTPase Rab7a and the role of this post-translation modification (PTM) in the function of Rab7a.
The Rab7a protein is acknowledged to be a key regulator of endosomal protein sorting having a number of effectors and being linked to many important physiological processes that occur at endosomes or down-stream of endosomes.
One of the effectors of Rab7a is the retromer complex - a conserved trimer of the Vps26, Vps29 and Vps35 proteins. In mammalian cells, retromer is required for efficient endosome to Golgi retrieval and also endosome to cell surface recycling. An interaction between Rab7a and retromer was first described from studies of Rab7 in enteric amoeba and, in mammalian cells, this association was determined to be important for the targeting/recruitment of retromer to endosomes. Subsequently, the SNX3 protein has been shown to also function with Rab7a in recruitment of retromer to endosomes.
The retromer complex has been shown to be important in the pathogenesis of Parkinson's disease and Alzheimer's disease (AD). Indeed, SNPs and variants of the Rab7a and SNX3 genes have been linked to late onset AD and a mutation in Rab7a that causes Charcot-Marie-Tooth disease (a peripheral neuropathy) does not associate with retromer. Thus the interaction between Rab7a and retromer is an important association from both a biological perspective and also from the perspective of understanding the origin of several neurodegenerative diseases.

Reviewer 1 Comments for the Author:
The authors report that phosphorylation of serine 72 is mediated by the NEK protein - a serine/threonine kinase and that this PTM impacts on the function of Rab7a with respect to its association with retromer and the further modification of Rab7a by palmitoylation.

© 2025. Published by The Company of Biologists under the terms of the Creative Commons Attribution License (https://creativecommons.org/licenses/by/4.0/).

The data presented will be of interest to the field, especially those working on retromer and endosomal protein sorting but I do have suggestions below for changes and experiments that I believe will significantly strengthen the manuscript.

Major points:
1. In Figure 2, I suggest to change the presentation of the immunofluorescence image data so that the colours used are not red and green. This can be problematic for individuals who are colour blind. I would advocate for using grey-scale for the Vps26a and Rab7a images and then have a colour merged panel with colours such as yellow and cyan instead of red and green. Why does the Rab7a localisation appear largely cytosolic - is that an expression issue, ie is Rab7a expressed transiently or through stable transfection?

Response: As per the reviewer's suggestion, we have updated the image using grey-scale, and we have modified the colour scheme in the merged images. Regarding the localization question raised by the reviewer, in our hands, when transiently expressed, we often find more Rab7A in the cytosolic fraction compared to endogenous Rab7A, especially when we are doing this type of rescue experiment.

2. Was the imaging performed blind? ie, did the person taking the images know what condition they were viewing? This is important because it is all too easy for unconscious bias to contaminate an experiment and impact the outcome when cells are 'selected' for imaging because they demonstrate the 'correct' phenotype.

Response: The imaging was performed blind.

3. Is there clear evidence that the S72A Rab7a mutant is actually properly folded etc? Is it known why the S72E mutant is apparently essentially non-functional? It seems slightly strange that the two mutations would have such different effects given that the serine at position 72 is likely to be at the surface of the protein (to be accessible to the kinase) and thus not likely to be critical for folding. Does the phosphorylation state of S72 impact the ability of Rab7a to hydrolyse GTP or interact with its GTPase activating protein (GAP) - TBC1D5? The S72 residue is not that far from a critical glutamine (at position 67) that is known to be important for GTP hydrolysis via an interaction with a Rab GAP.

Response: We are not aware of structural studies investigating the structure of Rab7A S72A mutant. However, we would argue that the protein is properly folded, as this mutant is expressed at levels similar to wild-type Rab7A, is membrane bound with a similar distribution to wild-type Rab7A (Figure S2A and B), and can still efficiently interact with RILP, PLEKHM1 and FYCO1 (Figure S1A - F). The fact that it interacts efficiently with other effectors also supports the conclusion that the mutant is GTP loaded. Although we do not have direct evidence that GTP hydrolysis by the mutant is comparable to wild-type Rab7A, we would argue that it is. If it were not, we would expect stronger interactions with effectors, which we show is not the case (Figure S1). The S72E mutant is not functional as it fails to interact with RGGTase, and as such is not prenylated. Without this modification, this mutant remains cytosolic, and can therefore not interact with effectors. This work was published by another group (Heo et al., 2018).

4. Could the graph shown in Figure 2H be redone with actual numbers rather than fold change? Also, the X-axis perhaps should include the signal obtained from imaging the 'wildtype' HEK293 cells which have not undergone any KO or transfection.

Response: In our opinion, the fold increase is the best way to present this data. Since the graph represents the fold increase in intensity compared to knockout cells, we chose to exclude the value of the wild-type cells. However, they are included in the image for reference.

5. It would be helpful if the GDP-locked Rab7a mutant (T22N) was also expressed to compare its effect on retromer localisation to the S72A mutant.

Response: In our experience, this mutant is not well expressed (Figure S2G). As such, we

© 2025. Published by The Company of Biologists under the terms of the Creative Commons Attribution License (https://creativecommons.org/licenses/by/4.0/).

believe that the prenylation mutant (Rab7A C205,207SA), which is cytosolic and therefore not functional, is a better negative control.

6. I would suggest that Figures 3 and 4 could be moved to supplemental or else combined into a single figure as they both show data from very similar experiments investigating the 'importance' of other kinases that apparently phosphorylate Rab7a.

Response: These figures have been merged into one figure (Figure 4). We think this is important data as it shows these kinases are not involved in retromer recruitment, even though they can phosphorylate Rab7A. As such, we feel that this figure should remain in the main body of the manuscript, and not be placed in the supplemental data.

7. The use of the biochemical assay (ie supernatant versus pellet) to investigate the membrane association of retromer is a sensible approach but I am slightly surprised at the variation in the appearance of the bands and also the relative levels of membrane association between the different experiments. For example, qualitatively, the bands shown in Figure 3A look rather different from those in Figures 3E, 4A and 4E. Levels of membrane associated Vps26 seem to vary quite a lot too - approx 40% membrane associated in 3B to 60% in 3F. The bands shown for Vps26 in Figure 3E suggest that (with DMSO) ~75% is membrane associated but the graph suggests a lower number. I recognise that these biochemical assays can be sometimes trickier than a microscopy-based assay but the variation is such that forming robust conclusions about the impact of mutations etc is potentially problematic.

Response: Figures 3A and 4A from the original manuscript have been removed from the current version of the manuscript. The difference observed from 3B to 3F in the previous version could be explained based on the cell line used. Figure 3F (now Figure 4D) are HeLa cells, while all other assays were performed in HEK293 cells. We attempted to knockout (KO) TBK1 in HEK293, but we were not obtain any KO clones. Although we understand the concern raised by the reviewer, we compare changes in the distribution per condition within the experiment, rather than between different experiments.

8. Is there any data to indicate what proportion of Rab7a is phosphorylated at S72? And also what proportion is palmitoylated? This is potentially important when the data from the Datta and Burd labs on Rab7a recruitment of retromer to endosomes is taken into consideration. Both of those labs employed in-vitro systems where the Rab7a may not be phosphorylated (or palmitoylated) but were able to show that, in vitro, Rab7a could support recruitment of retromer onto membranes. Thus, discussion of the importance of phosphorylation of S72 should incorporate findings from in-vitro systems where PTMs may not occur.

Response: We do not have any data that allows us to determine the percentage of Rab7A that is palmitoylated or phosphorylated at any given point in time. However, based on our experience, we would argue that the percentage of Rab7A that is palmitoylated is rather low as we capture a small fraction of Rab7A in our Acyl-RAC experiments. On the other hand, we do think that a significant amount is phosphorylated on serine 72, consistent with the involvement of several kinases involved in its phosphorylation. We agree with the reviewer that a discussion between in vitro finding versus our data is warranted, and this has now been included in the discussion. We have added a section in the discussion (Page 13).

9. Why was the loss of NEK on retromer localisation (ie Vps26) not investigated using immunofluorescence microscopy - as in Figure 2? This seems like an obvious approach to take.

Response: We find that the membrane assay system is simpler and faster to perform, and it enables us to measure the total amount of protein in each fraction. We attempted to use the same assay for Figure 2. However, in our hands, these rescue experiments with transient Rab7A expression are challenging. Enough cells must be transfected and express Rab7A so that we can observe an effect on a population of cells. However, to achieve this, we must use a larger amount of Rab7A plasmid and transfection reagent. We are hesitant to do this, as we believe excess amounts of Rab7A could lead to unintended effects. Therefore, we preferred to use the IF method in this case as in Figure 2.

© 2025. Published by The Company of Biologists under the terms of the Creative Commons Attribution License (https://creativecommons.org/licenses/by/4.0/).

10. Does loss of NEK7 impact on sortillin stability? This would be predicted (and possibly expected) if sortillin is not being efficiently retrieved from endosomes to the Golgi.

Response: We performed this experiment and found no changes in the stability of sortilin. Although it is reasonable to expect degradation, we hypothesize that NEK7 may play another role in the degradation pathway that we have not uncovered. Alternatively, the loss of NEK7 did affect lysosomal function (Figure 7D). As such, it is possible that the 6 hour cycloheximide chase was not long enough to observe degradation. However, longer incubation times can lead to cell death, so we did not extend the experiment to longer time points. Interestingly, this phenomenon is observed in Rab7A depleted or deleted cells (Rojas et al., 2008, Seaman et al., 2009, Yasa et al., 2020). In these cells, CI-MPR and sortilin are retained in endosomes, but are not degraded, as Rab7A plays a role in both the retrieval and degradative pathways.

11. Does mutation of S72 (or loss of NEK7) impact on the localisation of Glut1, a membrane protein that is dependent on retromer for its endosome to cell surface recycling?

Response: The reviewer does make an interesting comment, and this should be investigated in the future. However, we consider that this line of investigation is beyond the scope of this current work.

Minor points:
1. Perhaps it is now appropriate to no longer describe Rab7a-positive endosomes as 'late' endosomes. The 'early' and 'late' terminology no longer accurately describes the complexity or dynamism of the endosomal system and leaves the reader with the impression that endosomes comes in only two flavours - early and late. In my view, it is now time for the endosomal system to be viewed as a continuum of maturing compartments which are in a constant state of flux.

Response: We agree with the statement made by the reviewers. We have always agreed that the endosomal system is fluid and in constant flux. However, the field still uses these terms, so we are comfortable using this wording for simplicity.

2. I'm not sure why the Canuel 2008 paper is cited with respect to Rab7a coordinating retrieval from endosomes to the Golgi as that study is clearly focussed on the opposing roles of AP1 and retromer in mediating sortilin localisation. I would suggest moving that citation to more appropriate location in the text.

Response: We agree with the reviewer and have removed the Canuel reference from this section of the manuscript.

*****

Reviewer 2 Advance Summary and Potential Significance to Field:
General comments:

In this manuscript, Modica et al. extend on their previous work on Rab7 palmitoylation (Modica et al. JCS 2017). The authors establish a link between Rab7 phosphorylation at position S72 and palmitoylation at positions C83/C84. They demonstrate that Rab7 with an S72A mutation is less able to interact with VPS26A, consistent with their previous observations using Rab7 C83S/ C84S, and identify NEK7 as a Rab7 S72 kinase.

Reviewer 2 Comments for the Author:
The text is well-written, and figures are clearly presented. Some conclusions are well-supported by the data, however, some assays used in the manuscript require additional controls for the data to be properly interpreted; see specific points below.

Specific points:

Figure 1A, 5C, 7A, 7C – The BRET experiments require appropriate negative controls, such as non-interacting GFP- and RLuc- labelled proteins, to assess the background BRET in each

© 2025. Published by The Company of Biologists under the terms of the Creative Commons Attribution License (https://creativecommons.org/licenses/by/4.0/).

experimental system. This approach was used by the authors in their previous manuscript (Modica et al. JCS 2017, Figure 5A/B).

Response: We have added appropriate negative controls to the BRET experiments. In figure 1A, we have added RlucII-Rab1a and µ1-GFP10. In Figure 5C, we added CLN6-RlucII, and in Figure 7A, we added Vps41-GFP10 In Figure 7C, we added PMP70-RlucII.

Figure 2 – The data in this figure show a decrease in overall VPS26A intensity, however, there is still a punctate distribution in the images. Therefore, these data are more consistent with a loss of whole-cell levels of retromer, and not specific dissociation from endosomes as described in the text.

Response: Various groups over the years, including ours, have shown that depletion (siRNA) or deletion (CRISPR/Cas9) of Rab7A results in the displacement of retromer from the membrane, but not its degradation (Rojas et al., 2008; Seaman et al., 2009; Modica et al., 2017; Yasa et al., 2020). This finding is supported by membrane separation assays, which allows us to determine the amount of retromer subunits in the membrane and cytosolic fractions (Figure 6C).

Figure 3, 4 – These data are difficult to interpret without controls to show that the inhibition/ knockout of TBK1 and TAK1 is working correctly. Please could the authors include blots to show decreased phosphorylation of TBK1 (Figure 3) or TAK1 (Figure 4) substrates, and what effect the drugs/knockouts have on Rab7 S72 phosphorylation?

Response: We have added blots showing the knockout of TBK1 and TAK1 reduced Rab7A serine 72 phosphorylation (Figure S3A and B). As we have removed the inhibitor data from the current version of the manuscript, we did not include the pS72 blots for these results.

Figure 7C – It is surprising that the authors see such a robust BRET signal for LAMP1-sortilin when these proteins are not known to interact strongly (BRET = ~0.12 compared to a maximum of ~0.03 for Rab7/VPS26A). Therefore, it is particularly important to include appropriate negative/positive controls for this BRET pair and perform a titration similar to those in Figures 1A, 5C, 6A. Furthermore, Rab7 and NEK7 knockouts could substantially alter LAMP1/sortilin abundance and subcellular localisation, so additional controls should be included to account for possible indirect effects on BRET signal.

Response: One must be careful when comparing the BRET signal between two different interacting pairs. Since the BRET signal is inversely proportional to the distance between the luciferase and fluorescent protein, it is difficult to draw conclusions about the strength of the interaction between Rab7A/retromer (Vps26A) versus LAMP1/sortilin as the distance between the two tags could vary due to protein conformation and size. While we are measuring the propensity of the interaction between Rab7A and Vps26A, and how palmitoylation or phosphorylation of Rab7A affects this interaction, we are not directly measuring the interaction between LAMP1 and sortilin in Figure 7C, as this would require us to generate BRET titration curves such as the ones found in Figure 7A, and then calculate the $BRET_{50}$.
The BRET signal we observe ($BRET_{net}$) is known as a random collision and is meant to measure the amount of a protein in a specific compartment. LAMP1 is localized to late endosomes and serves to mark that compartment. An increase in the $BRET_{net}$ signal between sortilin-RlucII and LAMP1-YPet is interpreted as an increase in the amount of sortilin in the LAMP1 compartment. In $Rab7A^{KO}$ cells, sortilin is not efficiently retrieved back to the Golgi, and remains at the late endosome, but is not degraded (Yasa et al., 2020). The same is true for the mannose 6-phosphate receptor (Rojas et al., 2008). As lack of Rab7A results in more sortilin at late endosomes, we observe greater $BRET_{net}$ signal between sortilin and LAMP1.
This serves as the positive control. The variations of the amount of $BRET_{net}$ observed could not be explained by changes in the expression of one of the proteins as shown in Figure 7D. If changes in expression was occurring, the ratio of fluorescence and luminescence would also change, and this was not observed. We have also added a negative control with an

© 2025. Published by The Company of Biologists under the terms of the Creative Commons Attribution License (https://creativecommons.org/licenses/by/4.0/).

integral membrane protein known to localize to peroxisomes (PMP70). We found almost no signal between PMP70-RlucII and Lamp1-YPet, which was not changed between the parental and NEK7^{KO} cells.

Figure 7D – This assay also requires an appropriate negative control to assess the background signal, such as a sample without the Cathepsin L substrate or a sample with a Cathepsin L inhibitor.

Response: When this assay is performed, the fluorescence emitted by the cells without the Cathepsin L substrate is subtracted from the signal obtained with the substrate for each condition, in triplicate.
From the Methods Section;
Lysosomal Activity
To determine the activity of cathepsin L, wild-type, NEK7^{KO}, NEK7^{KO} expressing HA-NEK7 or HA-NEK7$_{K64M}$ and RAB7A^{KO} HEK293 cells were collected at a concentration of 3 × 10^{6} cells/ml of each cell type, then transferred to 96-well black wall plates in triplicate. Cells were then incubated with the Magic Red substrate for 60 min at 37°C protected from light. As cells settled to the bottom, they were gently resuspended by pipetting every 10-20 minutes to ensure that the Magic Red was evenly dispersed among all cells. The fluorescence intensity of the substrate was measured with a Tecan Infinite M1000 Pro plate reader (Tecan Group Ltd., Mannedorf, Switzerland) with the excitation and emission set at 592 nm and 628 nm, respectively. The average of non-stained sample fluorescence intensities was calculated for each sample and subtracted from the fluorescence reads of the Magic Red-stained samples to eliminate background fluorescence, and signals were standardized using Hoechst stain in each sample

Abstract/Discussion – The authors describe NEK7 as “the kinase required to phosphorylate Rab7”. However, Figure 5 shows that Rab7 maintains clear S72 phosphorylation even with the NEK7 knockout, so this statement should be altered to reflect the possibility of other kinases being involved.

Response: We have altered the statement to allow for other kinases being involved in the process. In the abstract, we changed the word “the” to “a”. In the discussion, we added the following sentence “Although we identified a role for NEK7 in this process, other kinases could also be involved”.

*****

Reviewer 3 Advance Summary and Potential Significance to Field:
The manuscript by Modica et al. investigates how phosphorylation of the late endosomal GTP-binding protein Rab7, specifically Serine 72, regulates Rab7 palmitoylation. The authors analyzed interaction of the phosphorylation-defective Rab7 mutant with the known interaction partner, the retromer complex. The authors have found that the “non-phosphorylatable Rab7A (Rab7AS72A) mutant does not efficiently interact with Vps26 subunit of retromer and fails to rescue retromer membrane recruitment when expressed in Rab7KO HEK293 cells. To uncover the mechanism, the authors analyzed the palmitoylation status of the phospho-defective Rab7 mutant and found that “Rab7AS72A is not efficiently palmitoylated." Neither TANK-binding kinase 1 (TBK1) nor TAK1 kinases (that have been shown to phosphorylate Rab7A) were found to regulate retromer membrane localization. However, the authors identified NEK7 kinase as a regulator of Rab7 phosphorylation and retromer localization. The authors also showed that NEK7 KO cells had reduced interaction between retromer and one of its cargos, Sortilin. Sortilin localization was likely more endolysosomal in NEK7 KO cells.

Reviewer 3 Comments for the Author:
The manuscript presents an interesting finding on regulation of Rab7, which is a key regulator of late endosomal trafficking. The study identifies NEK7 as one of the kinases that mediates Rab7 phosphorylation at S72 and thus acts as a regulator of Rab7 function. The authors show that Rab7AS72A interaction with retromer subunit Vps26 is reduced, and Vps26/retromer is displaced from vesicles. The mechanism behind the reduced effector binding of Rab7AS72A is unclear from

© 2025. Published by The Company of Biologists under the terms of the Creative Commons Attribution License (https://creativecommons.org/licenses/by/4.0/).

the presented data. Is this serine residue conserved in other Rab proteins and whether phosphorylation of serine residue at this position reported for other Rabs? Can the authors speculate from previous structural data on Rab-effector complexes, how this phosphorylation would change effector binding?

Response: Several of Rab GTPases contain a phosphorylation site, either a serine or threonine, in a similar position. For example, LRRK2 is known to phosphorylate Rab3, Rab8, Rab12, Rab29, Rab35 and Rab43. A recent publication addressed how phosphorylation could act as a binding switch (Tudorica et al., 2024). In brief, the structure of the Rab7A/rubicon interaction suggests that rubicon can interact with unphosphorylated Rab7A. On the other hand, the RH domain in Pacer, another Rab7A effector, is predicted to bind phosphorylated Rab7A. This phosphoswitch was found to be dependent on TBK1.

The authors should assess whether there is any change in GTP binding of Rab7AS72A, as compared to the WT protein. Is Rab7AS72A defective in binding to only retromer or to other Rab7 effectors, such as RILP, ORP1L and PLEKHM1? Any change in GTP binding would generally also result in a change in Rab's membrane localization.

Response: We did not observe any significant changes in the membrane distribution of Rab7AS72A when we performed a membrane separation assay experiment (Figure S1A and B). Although we do not have direct evidence of changes in the ability of Rab7AS72A to bind GTP, we argue that it does not affect GTP loading. As pointed out by the reviewer, changes in GTP binding should affect membrane localization (it doesn't in our hands), and the Rab7AS72A can still efficiently interact with RILP, PLEKHM1 and FYCO1 (Figure S1A - F). This supports the idea that GTP loading is not affected by the S72A mutation.

The authors should also analyse the localization and binding of Vps35 subunit of retromer, which is the direct binding partner of Rab7 in this complex. Retromer is a trimeric complex composed of the Vps35-Vps26 and Vps29 subunits. To conclude that the complete retromer complex is displaced from membranes, the authors should also show localization of the other subunits in the presence of Rab7 WT and mutants and interaction of the Rab7 S72A mutant with the complete retromer complex.

Response: It is well established that the retromer trimer is formed in the cytosol in mammalian cells, and that depletion/deletion of one subunit, results in the degradation of the others.
Therefore, we feel that using Vps26A as a representation of retromer is sufficient. Vps29 also plays a role in another complex (Retriever), so we have excluded this subunit from our analysis. Regarding Vps35, we have tried using this subunit in our BRET assays, however, we do not obtain a good signal. It could be due to a structural factor, such that the tags are farther apart, or it may be that the tagged subunit does not integrate the complex efficiently.

The authors show that the phosphorylation-defective Rab7 mutant (Rab7AS72A) continues to localize to membranes, however, the palmitoylation-defective Rab7C83,84S mutant translocates into the cytosol (Fig. S1 C-E). The author should provide an explanation that if Rab7AS72A is partially defective in palmitoylation, then how its membrane localization remain unchanged?

Response: In Figure S2 (previously Figure S1), the mutant used was the prenylation mutant (Rab7AC205,207S) which is known to be cytosolic, and not the palmitoylation mutant (Rab7AC83,84S). The palmitoylation mutant is indeed localized to endosomal membranes, as we have previously shown (Modica et al., 2017).

From figure 5A, it appears that a significant proportion of total Rab7 is phosphorylated at S72 under endogenous conditions, and that this is reduced upon Nek7 knockout. Where is this phosphorylation likely to occur, i.e., in the cytosol or on late endocytic membranes? Is Nek7 localized to late endosomes? The authors should also show the specificity of the anti-phospho Rab7 antibody used in Figure 5A.

© 2025. Published by The Company of Biologists under the terms of the Creative Commons Attribution License (https://creativecommons.org/licenses/by/4.0/).

Response: The specificity of the anti-phospho Rab7A antibody has been demonstrated in a number of recent publications (Malik et al., 2021 and Tudorica et al., 2024). We have also included our own data (Figure S2G). We found phosphorylation of wild-type myc-Rab7A, myc-Rab7AY183F and myc-Rab7AY183E, but not myc-Rab7AS72A. This suggests that the antibody can specifically recognize phosphorylated Rab7A on serine 72. Furthermore, the prenylation mutant, myc-Rab7AC205,207S , which is not membrane bound, is significantly less phosphorylated suggesting that Rab7A must be membrane bound in order to be modified. The C. elegans homologue of NEK7 does localize to endosomes (Jospeh et al., 2023), and NEK has been previously reported to interact with LAMP3 (Huttlin et al., 2021). Based on our own data, we argue that NEK7 is localized to endosomes as it interacts with Rab7A in live cells (Figure 5C).

The authors have not analyzed significance of Rab7 phosphorylation in late endosome/lysosome positioning and lysosomal cargo degradation. To better assess the significance of phosphorylation at this residue, the authors should assess whether Rab7AS72A can rescue the cargo trafficking function in the absence of Rab7, such as for cargo destined for degradation like EGFR or LDL degradation. Further, Rab7 regulates late endosome positioning in the perinuclear region by recruiting RILP-dynein-dynactin complex on late endosomes. Does expression of Rab7AS72A affect late endosome/lysosome positioning?

Response: We have included data on the degradation of EGF-488. As expected, cells lacking Rab7A are unable to degrade EGF-488, while parental HEK293 cells can (Figure 3). However, degradation is restored by expressing either myc-Rab7A or myc-Rab7AS72A, but not by expressing myc-Rab7AC205,207S. This suggests that phosphorylation on serine 72 is not required for this pathway. RILP and FYCO1 are able to bind to Rab7A S72A (Figure S1) as efficiently as wild-type Rab7A. As these proteins are involved in lysosomal postioning, we did not investigate whether this mutant affects endosome/lysosome positioning.

The authors show that NEK7 mediates Rab7 phosphorylation at S72. Is there endocytic cargo degradation defects in NEK7 KO cells and whether the same can be rescued by overexpression of Rab7WT?

Response: As we did not observe any changes in endocytic degradation in cells expressing Rab7A S72A (Figure 3), we did not investigate whether NEK7 plays a role in this process.

Below, I list some of the concerns in specific figures, as well as my suggestions to strengthen the study's major conclusions:

1) Fig. 1C: The mobility of palmitoylation-defective Rab7C83,84S is shifted upward in both pulldown and WCL. The same upward mobility shift is not evident in Rab7AS72A. What is the reason behind the upward mobility shift of Rab7C83,84S?

Response: This is an observation we have previously observed (Modica et al., 2017). It could be due to changes in other post-translational modifications, such as ubiquitination. However, we have never directly investigated the cause of this mobility shift.

2) The localization data shown in Figure 2 (A-G) is confusing. Myc Rab7A constructs, both WT and all the mutants, except Y183F, appear to be very cytosolic. Typically, Rab7a WT is expected to be on membrane-bound vesicles. This is not evident in Fig. 2A.

Response: We agree with the reviewer and have modified the representative images in Figure 2. Although we still observe some Rab7A expression in the cytosol, it is not as significant as in the previous version of the figure. The issue arises from the use of transient expression. When using this technique, the cell often over expresses the protein, resulting in the excessive localization to the cytosol.

3) In the same set of panels, i.e., Figure 2 (A-G), it is unclear why Vps26 total intensity is reduced in cells lacking functional Rab7. Shouldn't it be that Vps26 shifts from vesicles to the cytosol in cells with reduced Rab7 function?

© 2025. Published by The Company of Biologists under the terms of the Creative Commons Attribution License (https://creativecommons.org/licenses/by/4.0/).

Response: If we perform a membrane separation assay on Rab7AKO, we observe a shift of retromer from the membrane fraction to the cytosolic fraction (Figure 6C and D, and Yasa et al., 2020) as the reviewer points out. Ideally, this experiment would have been performed using a membrane separation assay protocol. However, we were technically unable to use this method for the Rab7A^{KO} cells for the rescue. To rescue the cells, we transiently express myc- Rab7A and the various mutants. In order to observe a phenotype, a sufficient number of cells need to express the protein at a high enough level. In our hands, this was difficult as expressing more myc-Rab7A in cells affected cell behaviour. We therefore chose to perform this experiment using immunofluorescence (IF).

4) To strengthen the findings of Fig. 7C, the authors should also show images and measure Sortilin colocalization with LAMP1-positive compartments under different conditions.

Response: We have not had much success with sortilin antibodies for immunofluorescence labelling, and have, previously relied on over-expressed sortilin-myc. Rather than performing this experiment as we have in the past, we chose to use a BRET technique. In our opinion, there are several advantages to this method compared to immunofluorescence (IF) microscopy. This method is more sensitive than IF, which enables us to use significantly less plasmid, and consequently express much less protein. Since the data is collected using a plate reader, there is no bias, which can occur when capturing images.

Minor concerns:
-Pg 3 and 4: Mutant RlucII-Rab7A Y183F is miswritten as RlucII-Rab7A Y183E in the
- Fig 1B- myc Rab7 is labeled on X axis while RlucII-Rab7A constructs have been used for transfections.
-Fig S1 C, D, and E: scale bar missing
-Page 7: 10 ng- symbol is incorrect
-Page 11: Typo in Rab7A s72 phosphorylation.
-Page 13: Membrane Separation Assay- what does the statement centrifuged 5' mean?

Response: We thank the reviewer for pointing out these errors. They have been corrected.

Second decision letter

MS ID#: JOCES/2024/262177

MS TITLE: Phosphorylation on serine 72 modulates Rab7A palmitoylation and retromer recruitment

AUTHORS: Graziana Modica, Laura Tejeda-Valencia, Etienne Sauvageau, Seda Yasa, Juliette Maes, Olga Skorobogata, and Stephane Lefrancois
ARTICLE TYPE: Research Article

I am happy to tell you that your manuscript has been accepted for publication in Journal of Cell Science, pending standard publication integrity checks.

Reviewer 1

Advance summary and potential significance to field

See previous review.

Comments for the author

The authors have revised the manuscript to address many of the issues I raised previously.

© 2025. Published by The Company of Biologists under the terms of the Creative Commons Attribution License (https://creativecommons.org/licenses/by/4.0/).

I'm surprised that in their response to point 5 they regard the prenylation mutant as a better control than the GDP-locked mutant. Given the nature of Rab proteins are molecular switches, using the GDP- or GTP-locked mutants as controls for experiments to address the impact of specific mutations on function has become standard practice. Certainly the T22N mutant is less well expressed than other Rab mutants but it has been used extensively by other labs in published studies and was used in studies focussed on the role of Rab7 in regulating retromer recruitment. The prenylation mutant is, in my view, inferior to the T22N mutant as a control and comparator. The response to points 10 and 11 are also surprising. Loss of NEK would, if the conclusions of the study are true, be predicted to impact retromer function and thus would likely lead to reduced cycling of retromer cargo proteins but would not necessarily perfectly phenocopy the loss of Rab7 function as Rab7 operates (to regulate membrane fusion etc) on endolyosomes/lysosomes where retromer is not present. If the authors regarded the stability of sortilin as worth investigating, then why would Glut1 localization be beyond the scope of the study?

Reviewer 2

Advance summary and potential significance to field

In their revisions the authors have addressed all of the points raised either through further experiments or changes to the text. This is an interesting and important study.

Comments for the author

The paper is now suitable for publication.

Reviewer 3

Advance summary and potential significance to field

The manuscript by Modica et al. investigates how phosphorylation of the late endosomal GTP-binding protein Rab7, specifically Serine 72, regulates Rab7 palmitoylation. The authors have found that the “non-phosphorylatable Rab7A (Rab7AS72A) mutant does not efficiently interact with Vps26 subunit of retromer and fails to rescue retromer membrane recruitment when expressed in Rab7KO HEK293 cells. Neither TANK-binding kinase 1 (TBK1) nor TAK1 kinases (that have been shown to phosphorylate Rab7A) were found to regulate retromer membrane localization.
However, the authors identified NEK7 kinase as a regulator of Rab7 phosphorylation and retromer localization. The authors also showed that NEK7 KO cells had reduced interaction between retromer and one of its cargos, Sortilin. Sortilin localization was likely more endolysosomal in NEK7 KO cells.

Comments for the author

The authors have satisfactorily addressed all of my concerns; specifically, the authors have clarified the role of Rab7 serine 72 phosphorylation in endocytic cargo trafficking and also investigated the ability of this mutant in binding to other Rab7 effectors.

© 2025. Published by The Company of Biologists under the terms of the Creative Commons Attribution License (https://creativecommons.org/licenses/by/4.0/).
